# The monothiol glutaredoxin GrxD is essential for sensing iron starvation in *Aspergillus fumigatus*

**DOI:** 10.1371/journal.pgen.1008379

**Published:** 2019-09-16

**Authors:** Matthias Misslinger, Mareike Thea Scheven, Peter Hortschansky, Manuel Sánchez López-Berges, Katharina Heiss, Nicola Beckmann, Thomas Heigl, Martin Hermann, Thomas Krüger, Olaf Kniemeyer, Axel A. Brakhage, Hubertus Haas

**Affiliations:** 1 Institute of Molecular Biology, Biocenter, Medical University of Innsbruck, Innsbruck, Austria; 2 Department of Molecular and Applied Microbiology, Leibniz Institute for Natural Product Research and Infection Biology—Hans Knöll Institute (HKI), Jena, Germany; 3 Institute of Microbiology, Friedrich Schiller University Jena, Jena, Germany; 4 Department of Anaesthesiology and Critical Care Medicine, Medical University of Innsbruck, Innsbruck, Austria; Universität Marburg, UNITED STATES

## Abstract

Efficient adaptation to iron starvation is an essential virulence determinant of the most common human mold pathogen, *Aspergillus fumigatus*. Here, we demonstrate that the cytosolic monothiol glutaredoxin GrxD plays an essential role in iron sensing in this fungus. Our studies revealed that (i) GrxD is essential for growth; (ii) expression of the encoding gene, *grxD*, is repressed by the transcription factor SreA in iron replete conditions and upregulated during iron starvation; (iii) during iron starvation but not iron sufficiency, GrxD displays predominant nuclear localization; (iv) downregulation of *grxD* expression results in de-repression of genes involved in iron-dependent pathways and repression of genes involved in iron acquisition during iron starvation, but did not significantly affect these genes during iron sufficiency; (v) GrxD displays protein-protein interaction with components of the cytosolic iron-sulfur cluster biosynthetic machinery, indicating a role in this process, and with the transcription factors SreA and HapX, which mediate iron regulation of iron acquisition and iron-dependent pathways; (vi) UV-Vis spectra of recombinant HapX or the complex of HapX and GrxD indicate coordination of iron-sulfur clusters; (vii) the cysteine required for iron-sulfur cluster coordination in GrxD is *in vitro* dispensable for interaction with HapX; and (viii) there is a GrxD-independent mechanism for sensing iron sufficiency by HapX; (ix) inactivation of SreA suppresses the lethal effect caused by GrxD inactivation. Taken together, this study demonstrates that GrxD is crucial for iron homeostasis in *A*. *fumigatus*.

## Author summary

*Aspergillus fumigatus* is a ubiquitous saprophytic mold and the major causative pathogen causing life-threatening aspergillosis. To improve therapy, there is an urgent need for a better understanding of the fungal physiology. We have previously shown that adaptation to iron starvation is an essential virulence attribute of *A*. *fumigatus*. In the present study, we characterized the mechanism employed by *A*. *fumigatus* to sense the cellular iron status, which is essential for iron homeostasis. We demonstrate that the transcription factors SreA and HapX, which coordinate iron acquisition, iron consumption and iron detoxification require physical interaction with the monothiol glutaredoxin GrxD to sense iron starvation. Moreover, we show that there is a GrxD-independent mechanism for sensing excess of iron.

## Introduction

Iron is an essential trace element for almost all organisms in all kingdoms of life. On the other hand, iron excess is toxic. Therefore, to maintain cell homeostasis, the balance between iron uptake and iron consumption has to be tightly regulated.

Previous studies have shown that iron homeostasis in the pathogenic mold *Aspergillus fumigatus* is mainly regulated by two transcription factors, SreA, the repressor of siderophore biosynthesis and reductive iron assimilation [1], and HapX, which is a repressor of iron-consuming pathways and activator of iron acquisition [2]. Moreover, HapX is essential for adaptation to iron excess. When iron concentrations increase, HapX changes its function from a repressor to an activator of iron-consuming and detoxifying pathways to avoid iron toxicity. Consequently, HapX is crucial for adaptation to both iron starvation (-Fe) and high iron concentrations (hFe), i.e. lack of this regulator causes growth defects under -Fe as well as hFe [3]. Notably, both the -Fe and hFe functions of HapX require the HapB/HapC/HapE CCAAT-binding complex (CBC) as a DNA binding platform [4].

SreA and HapX are interconnected in a feedback-loop [5]: Expression of *sreA* is repressed by HapX during -Fe [2] and, in turn, *hapX* expression is repressed by SreA under iron sufficiency/excess [1]. Moreover, HapX induces *sreA* expression in response to iron.

Fungal iron sensing has been studied most intensively so far in the yeasts *Saccharomyces cerevisiae* and *Schizosaccharomyces pombe* [6,7]. Remarkably, there is little similarity with respect to transcriptional iron regulation between *S*. *cerevisiae* and *A*. *fumigatus*. Despite the fact that both, HapX and SreA are conserved in most ascomycetes, *S*. *cerevisiae* lacks classical homologs of SreA and HapX. In this yeast, adaptation to iron starvation is mainly mediated by two paralogous transcription factors, termed Aft1 and Aft2 [8–10]. Adaptation to hFe by transcriptional activation iron detoxification is mediated by the bZIP transcription factor Yap5 [11]. Nevertheless, *S*. *cerevisiae* Yap5 and HapX show similarities. Both transcription factors are essential for iron detoxification by activation of vacuolar iron deposition. Moreover, they share a highly conserved cysteine-rich region (CRR) that is crucial for this function and which has been shown to coordinate a [2Fe-2S] cluster in Yap5 [3,12]. In contrast to HapX, however, Yap5 has no function during iron starvation. *S*. *pombe* employs a homolog of SreA, termed Fep1 [13] and a regulator displaying similarity with HapX, termed Php4 [14]. Similar to HapX, Php4 acts as repressor of iron-consuming functions during iron starvation, but in contrast to HapX it is not involved in activation of iron detoxification. Taken together, *S*. *cerevisiae*, *S*. *pombe* and *A*. *fumigatus* show significant differences with regard to the employed iron-regulatory transcription factors and the molecular mechanisms of iron sensing in *A*. *fumigatus* are largely uncharacterized.

In both *S*. *cerevisiae* and *S*. *pombe*, the cytosolic monothiol glutaredoxins Grx3/4 respectively Grx4 have been shown to be involved in iron sensing [15,16] and coordination and transport of [2Fe-2S] clusters. These proteins contain a thioredoxin (Trx)-like domain, for which a canonical reductase activity has been excluded [17], and a glutaredoxin (Grx) domain comprising a highly conserved CGFS motif. Coordination of [2Fe-2S] clusters is performed via the cysteine residue of the CGFS motif and two glutathione residues, which leads to dimerization of these monothiol glutaredoxins [18–20].

In the current study, we characterized the role of the cytosolic monothiol glutaredoxin of *A*. *fumigatus* (Afu2g14960), designated GrxD. We demonstrate that GrxD is essential for iron sensing by the iron-responsive transcription factors HapX and SreA, particularly for signaling iron starvation conditions. The study revealed both similarities and differences to iron sensing in other fungal species.

## Results

### GrxD is essential for *A*. *fumigatus*

**Fig 1:**
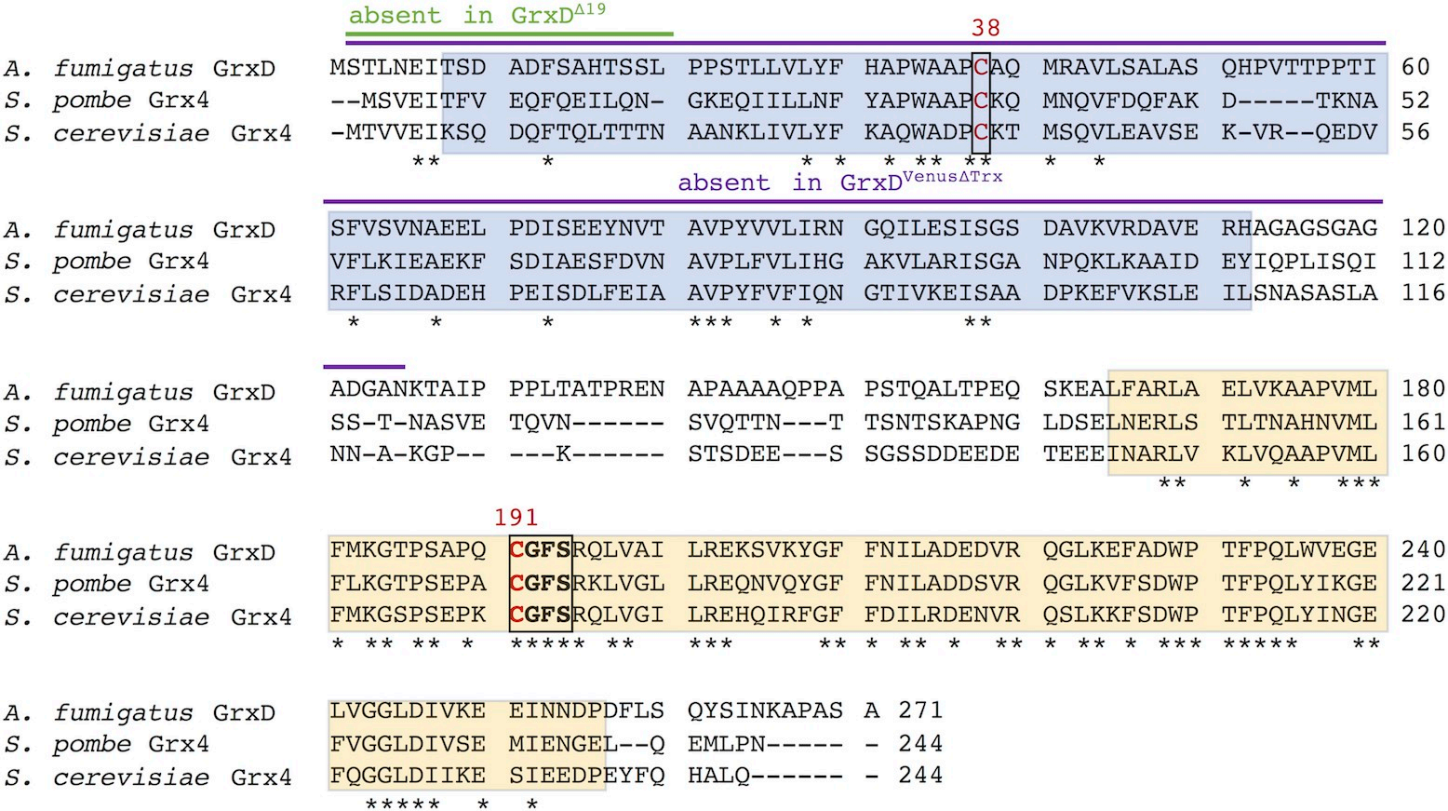
Alignment of GrxD homologs from *A*. *fumigatus*, *S*. *cerevisiae*, and *S*. *pombe*. Trx-like and Grx domains are highlighted in blue and yellow, respectively. The strictly conserved monothiol glutaredoxin specific CGFS motif and the conserved single cysteine residue in the Trx domain are framed. Identical amino acids in all three proteins are marked by asterisks. Amino acids, which are absent in truncated protein versions GrxD^Δ19^ or GrxD^VenusΔTrx^, are indicated by bars in green or purple, respectively.

Protein BLAST searches identified the *A*. *fumigatus* homolog, termed GrxD, of *S*. *cerevisiae* Grx3/4 and *S*. *pombe* Grx4, respectively. Alignment of GrxD homologs demonstrated high conservation, even between distantly related species (Figs 1 and S1). Compared to the Trx-like domain, the Grx domain shows significantly higher conservation including the [2Fe-2S] cluster coordinating CGFS motif.

**Fig 2:**
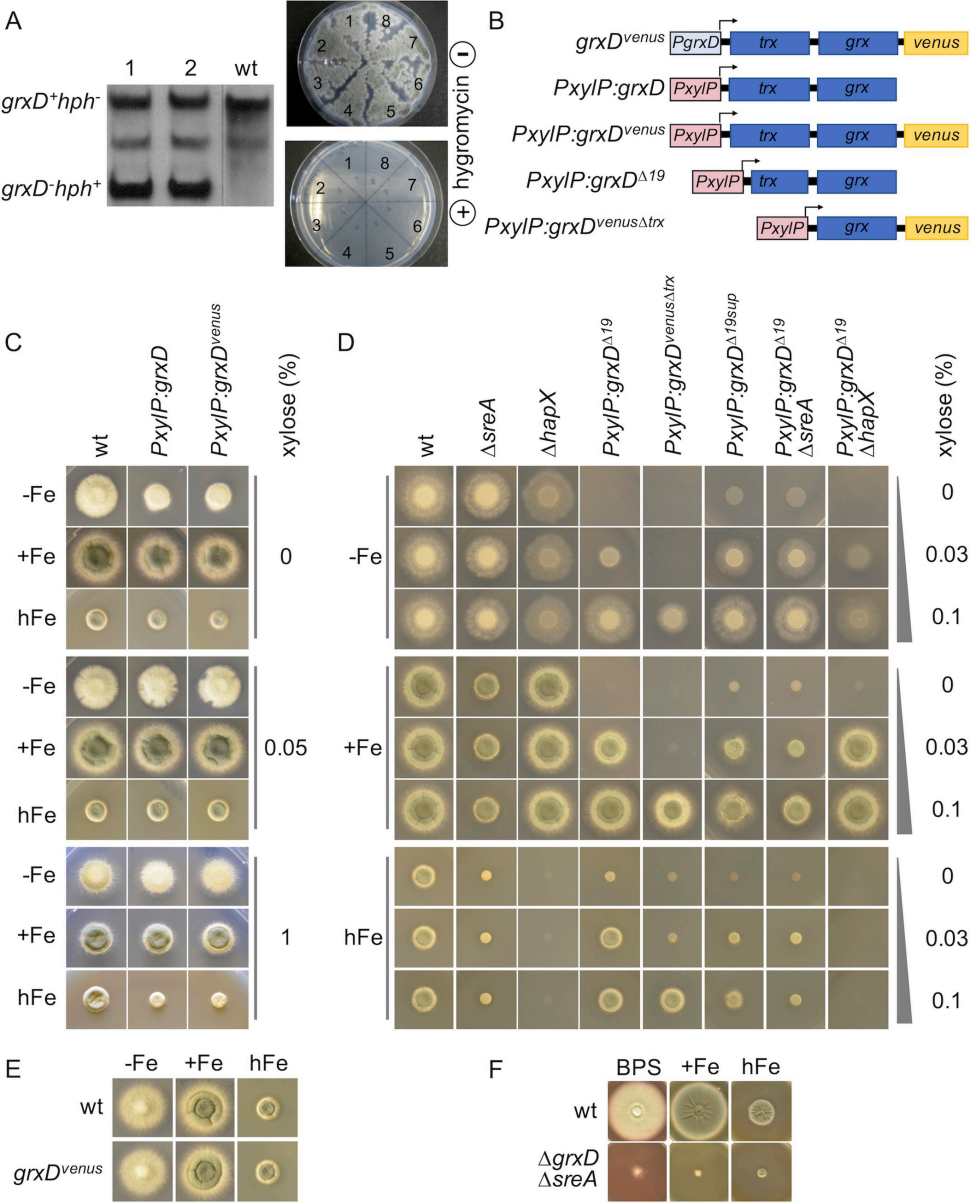
GrxD is essential, influences iron-dependent growth and SreA inactivation suppresses the lethal effect of GrxD inactivation. Strains were grown for 48 h at 37°C in minimal medium, with exception of F, where complex medium was used. In general, media contained 1% glucose with exception of media containing 1% xylose, which leads to maximal activation of the *PxylP* promoter. -Fe, +Fe, hFe media contained no, 0.03 mM and 10 mM iron, respectively. BPS media contained 0.2 mM of the ferrous-iron specific chelator bathophenanthroline disulfonate to generate iron starvation in complex media. (A) Heterokaryon rescue: Exemplary Southern blot analysis showing heterokaryosity of two independent transformants because of the presence of wt as well as *ΔgrxD* alleles; eight independent heterokaryotic transformants were able to grow under non-selective conditions but failed to grow in the presence of hygromycin indicating essentiality of *grxD*. (B) Schematic overview of generated strains for *PxylP*-mediated conditional expression of *grxD* variants indicating truncations and Venus-tagging (C) *PxylP*-mediated downregulation of full-length GrxD (*PxylP*:*grxD*) and full-length Venus-tagged GrxD (*PxylP*:*grxD*^*venus*^) decreased growth during -Fe. Overexpression decreased growth during hFe (D) *PxylP*-mediated downregulation of N-terminal truncation of GrxD (*PxylP*:*grxD*^*Δ19*^) and, even more pronounced, truncation of the Trx domain (*PxylP*:*grxD*^*venusΔtrx*^) blocked growth during iron starvation, which was rescued by iron supplementation. A spontaneous suppressor mutation (*PxylP*:*grxD*^*Δ19sup*^) as well as inactivation of SreA (*PxylP*:*grxD*^*Δ19*^/*ΔsreA*) rescued the growth defect of downregulation of N-terminal truncated GrxD (*PxylP*:*grxD*^*Δ19*^) during -Fe and +Fe. (E) C-terminal Venus-tagging (*PxylP*:*grxD*^*venus*^) did not affect the function of GrxD. (F) Inactivation of SreA (*ΔgrxD/ΔsreA*) suppressed lethality of lack of GrxD (*ΔgrxD*).

To investigate GrxD function in *A*. *fumigatus*, we aimed to delete the *grxD* gene via replacement by a hygromycin resistance-conferring cassette (*hph*) (S2 Fig). Several attempts were unsuccessful, indicating that *grxD* is an essential gene, which we proved by heterokaryon rescue [21]. In short, this technique is based on the fact that *A*. *fumigatus* cells contain multiple nuclei. The fungal transformation procedure usually targets only the genome of one nucleus leading to heterokaryosity, in our case *grxD*^*+*^*hph*^*-*^ (wt; containing *grxD* but lacking *hph*) nuclei and *grxD*^*-*^*hph*^*+*^ (*ΔgrxD;* lacking *grxD* but containing *hph* conferring hygromycin resistance) nuclei, which was proven by Southern blot analysis (Fig 2A). During conidiation, nuclei are separated since conidia contain only a single nucleus. Conidia of eight heterokaryotic transformants were able to grow under non-selective conditions but not in the presence on hygromycin (Fig 2A), demonstrating the inability of *ΔgrxD* (*grxD*^*-*^*hph*^*+*^) conidia to grow; i.e. *grxD* is an essential gene.

Due to the lethality of *grxD* deletion, we generated strains, in which *grxD* is under the control of the xylose-inducible *xylP* promoter (*PxylP*, [22]). These strains were generated without and with C-terminal tagging of GrxD with the yellow fluorescent protein derivative Venus, yielding strains *PxylP*:*grxD* and *PxylP*:*grxD*^*venus*^, respectively (Fig 2B). *PxylP* displays xylose concentration-dependent activation. Without xylose supplementation, activity of this promoter is very low, i.e. expression of essential genes under this promoter in *A*. *fumigatus* led to the inability to grow [23]. Although we proved that *grxD* is essential (Fig 2A), strains *PxylP*:*grxD* and *PxylP*:*grxD*^*venus*^ were able to grow without xylose-induction on solid minimal medium (Fig 2C). This indicates that very low expression is sufficient to support growth. Nevertheless, we observed growth deficiencies under iron starvation, which were ameliorated with increasing iron concentrations (Fig 2C), which indicates a role of GrxD in iron homeostasis. Overexpression of *grxD* with and without *venus*-tagging decreased growth under excess iron, but not under iron starvation or iron replete conditions (Fig 2C), indicating that a surplus of GrxD impedes adaptation to high iron conditions.

### The Trx domain is not essential for growth

To further analyze GrxD function, we generated *A*. *fumigatus* mutants producing *PxylP*-driven GrxD variants lacking either the 19 N-terminal amino acids (strain *PxylP*:*grxD*^*Δ19*^) or the whole Trx domain (*PxylP*:*grxD*^*venusΔtrx*^, Fig 1), whereby in the latter strain GrxD was C-terminally tagged with Venus (Fig 2B). Under non-inducing conditions (without xylose), truncation of 19 N-terminal amino acids or truncation of the complete Trx domain, respectively, blocked growth during iron starvation and iron sufficiency (Fig 2D). Growth of both mutant strains was rescued by xylose supplementation, whereby the strain expressing the Trx domain lacking GrxD required higher xylose supplementation indicating lower activity. Important to note, C-terminal tagging with Venus did not affect function of GrxD, at least judged by growth ability (Fig 2E).

The fact that, in contrast to strains *PxylP*:*grxD* and *PxylP*:*grxD*^*venus*^, strains *PxylP*:*grxD*^*Δ*19^ and *PxylP*:*grxD*^*venusΔtrx*^ were unable to grow in -Fe conditions under non-induced conditions indicates that truncation of the N-terminal 19 amino acids or, even more pronounced, the truncation of the Trx domain decreases activity of GrxD. This might be due to decreased protein stability or hampered function. Nevertheless, under xylose-inducing conditions, all strains were able to grow under all conditions, which indicates that in contrast to the whole GrxD protein, the Trx domain is not essential for growth, at least when overexpressed. Consequently, the Grx domain is likely essential for growth.

### Iron supplementation partially rescues GrxD deficiency

As shown above, N-terminal truncated GrxD versions (*PxylP*:*grxD*^*Δ*19^ and *PxylP*:*grxD*^*venusΔtrx*^) were not able to grow at non-inducing conditions during iron starvation or iron sufficiency (Fig 2D). However, high iron supplementation partially rescued the growth of these strains at non-inducing conditions (Fig 2D). These data indicate that GrxD is involved in iron homeostasis with an important role especially during iron starvation. This is in agreement with decreased growth of strains with down-regulated GrxD, without and with C-terminal Venus-tagging, under iron starvation but not iron sufficiency and iron excess (Fig 2C).

### Inactivation of SreA suppresses the lethality caused by lack of GrxD

Occasionally, cultivation of *PxylP*:*grxD*^*Δ19*^ conidia on plates resulted in suppressor mutants. We characterized one of these mutant strains, termed *PxylP*:*grxD*^*Δ19sup*^, in more detail. In contrast to *PxylP*:*grxD*^*Δ19*^, *PxylP*:*grxD*^*Δ19sup*^ was able to grow without xylose-induction under iron starvation and iron sufficiency (Fig 2D). Under 0.1% xylose-inducing conditions, *PxylP*:*grxD*^*Δ19sup*^ displayed a similar radial growth under iron starvation but decreased growth under iron sufficiency and high iron conditions compared to *PxylP*:*grxD*^*Δ19*^ (Fig 2D). These results indicated that the suppressor mutation present in this strain leads to a defect in adaptation to iron excess.

Northern analysis revealed an additional *sreA* transcript as well as de-repression of *hapX* and *mirB* (encoding a siderophore transporter) during iron sufficiency in strain *PxylP*:*grxD*^*Δ19sup*^ compared to *PxylP*:*grxD*^*Δ19*^ (S3A Fig). These results suggested that the suppressor mutation affects the function of SreA, which has previously been shown to repress transcription of these two genes [1]. PCR amplification analyses of the *sreA* locus (S3B and S3F Fig) followed by rapid amplification of cDNA ends (3´-RACE) and nucleotide sequencing (S3C and S3D Fig) revealed that the suppressor mutation caused a chromosomal rearrangement (S3E Fig), which results in truncation of SreA within the DNA-binding region.

To independently confirm the genetic interaction between *grxD* and *sreA*, the *sreA* gene was deleted in a *PxylP*:*grxD*^*Δ19*^ background. This mutant, *PxylP*:*grxD*^*Δ19*^/*Δ sreA*, displayed the same growth pattern as *PxylP*:*grxD*^*Δ19sup*^ (Fig 2D), which affirms that *sreA* loss-of-function rescues the growth defect caused by down-regulation of *grxD* during iron starvation and sufficiency.

To analyze whether inactivation of SreA rescues growth only in response to downregulation of GrxD (*PxylP*:*grxD*^*Δ19*^/*ΔsreA* at non-inducing conditions) or also complete lack of GrxD, we aimed to delete the *grxD* gene in a *ΔsreA* background. In contrast to wt background (see above), this approach was successful. Compared to wt, the *ΔgrxD*/*ΔsreA* strain displayed severely decreased radial growth under iron starvation, iron sufficiency and iron excess, but it was viable (Fig 2F).

SreA is the repressor of iron uptake and SreA inactivation results in increased iron acquisition [1]. Consequently, the identified genetic interaction between *grxD* and *sreA*, together with the rescue of growth of the *PxylP*:*grxD*^*Δ19*^ strain under non-inducing conditions by high iron supplementation (Fig 2D), indicate that lack of GrxD results in iron shortage, possibly caused by the requirement of GrxD for sensing iron starvation.

### Iron starvation increases *grxD* expression and promotes nuclear localization

**Fig 3:**
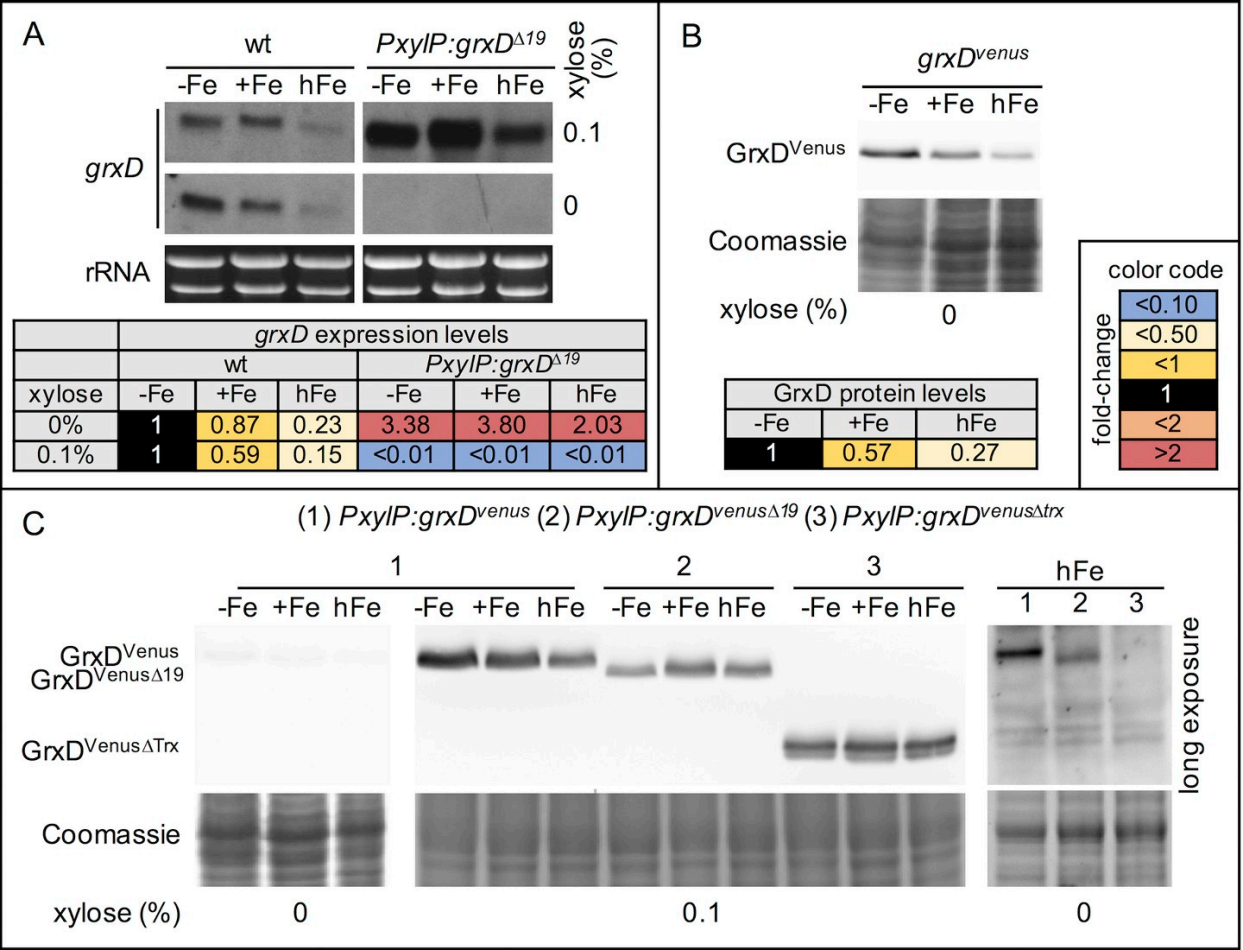
Expression of *grxD* is regulated by iron availability and controlled by xylose when expressed under control of the *PxylP* promoter. (A) For Northern analysis, RNA from wt and *PxylP*:*grxD*^*Δ19*^ was isolated after growth for 20 h at 25°C in inducing conditions and subsequent growth under non-inducing conditions at -Fe, +Fe and hFe for 20 h at 37°C. As a control, inducing conditions were maintained (0.1% xylose). (B,C) For Western blot analysis, *grxD*^*venus*^, *PxylP*:*grxD*^*venus*^ and *PxylP*:*grxD*^*venusΔtrx*^ were harvested after growth for 16 h at -Fe, +Fe or hFe with the indicated xylose concentrations. Loading of equal protein amounts was confirmed by Coomassie staining; Venus-tagged GrxD or variants thereof were detected with a mouse α-GFP antibody. Western blot analysis of GrxD^venusΔtrx^ under non-inducing conditions is only shown for hFe as this is the only condition in which *PxylP*:*grxD*^*venusΔtrx*^ is able to grow. For quantification of Northern and Western blot data (Tables), images were analyzed using ImageJ. Northern blot signals were first normalized to the respective rRNA and subsequently to the reference strain, whereby genes repressed by iron (*mirB*, *hapX*, *grxD*) were normalized to the reference strain grown under iron starvation, while genes induced by iron (*sreA*, *cccA*) were normalized to the reference strain grown under iron sufficiency. GrxD^Venus^ protein levels were first normalized to total protein and subsequently to the reference strain grown under iron starvation.

To monitor endogenous and *PxylP*-controlled *grxD* expression, we performed Northern analysis. In wt *grxD* transcript levels decreased with increasing iron supplementation (Fig 3A). In *PxylP*:*grxD*^*Δ19*^, *grxD* expression was highly induced under xylose-induced conditions and decreased below detection limit upon xylose withdrawal demonstrating functionality of *PxylP*-mediated conditional *grxD* expression (Fig 3A).

In agreement with wt *grxD* transcript levels, Western blot analysis demonstrated that Venus-tagged GrxD (GrxD^Venus^) protein levels decreased with increasing iron availability when *grxD* was expressed from the endogenous promoter (strain *grxD*^*venus*^; Fig 3B). Under control of the *xylP* promoter, the protein level of Venus-tagged full-length GrxD (GrxD^Venus^, strain *PxylP*:*grxD*^*venus*^) was highly decreased under non-inducing compared to inducing conditions (Fig 3C). Interestingly, hFe conditions slightly decreased the GrxD^Venus^ protein level under xylose-inducing conditions, which indicates an influence of iron on *xylP* promoter activity or on the *grxD* transcript stability.

To analyze protein levels of the GrxD variant lacking the 19 N-terminal amino acids (GrxD^Δ19^), we generated a strain in which C-terminally Venus-tagged GrxD^Δ19^ is under the control of the *xylP* promoter (strain *PxylP*:*grxD*^*venusΔ19*^). This strain showed identical growth compared to the untagged version *PxylP*:*grxD*^*Δ19*^ (S4 Fig). Compared to GrxD^Venus^, the protein levels of the Venus-tagged GrxD variants lacking the 19 N-terminal amino acids (GrxD^VenusΔ19^, strain *PxylP*:*grxD*^*venusΔ19*^) or the Trx domain (GrxD^VenusΔTrx^, strain *PxylP*:*grxD*^*venusΔtrx*^) were slightly decreased under inducing conditions. Remarkably, under steady-state, non-inducing, high iron conditions (Fig 3C), truncation of the 19 N-terminal amino acid residues (Grx^Venus*Δ19*^) decreased the protein level compared to GrxD^Venus^ although not as much as truncation of the entire Trx domain (Grx^VenusΔTrx^). Due to the use of the same promoter, these data indicate higher protein stability of GrxD^Venus^ compared to the truncated versions. These results most likely provide the explanation for the lack of growth of strains *PxylP*:*grxD*^*venusΔ19*^ and *PxylP*:*grxD*^*venusΔtrx*^ during iron starvation and sufficiency under non-inducing conditions (Figs 2D and S4) in contrast to strain *PxylP*:*grxD*^*venus*^ (Fig 2C).

Subcellular localization of Venus-tagged GrxD was determined by fluorescence. To visualize the nucleus, we expressed a gene encoding histone H2A tagged with monomeric red fluorescence protein (H2A^mRFP^) in recipient strains *PxylP*:*grxD*^*venus*^ and *PxylP*:*grxD*^*venusΔtrx*^ (yielding strains *PxylP*:*grxD*^*venus*^/*H2A*^*mRFP*^ and *PxylP*:*grxD*^*venusΔtrx*^/*H2A*^*mRFP*^).

**Fig 4:**
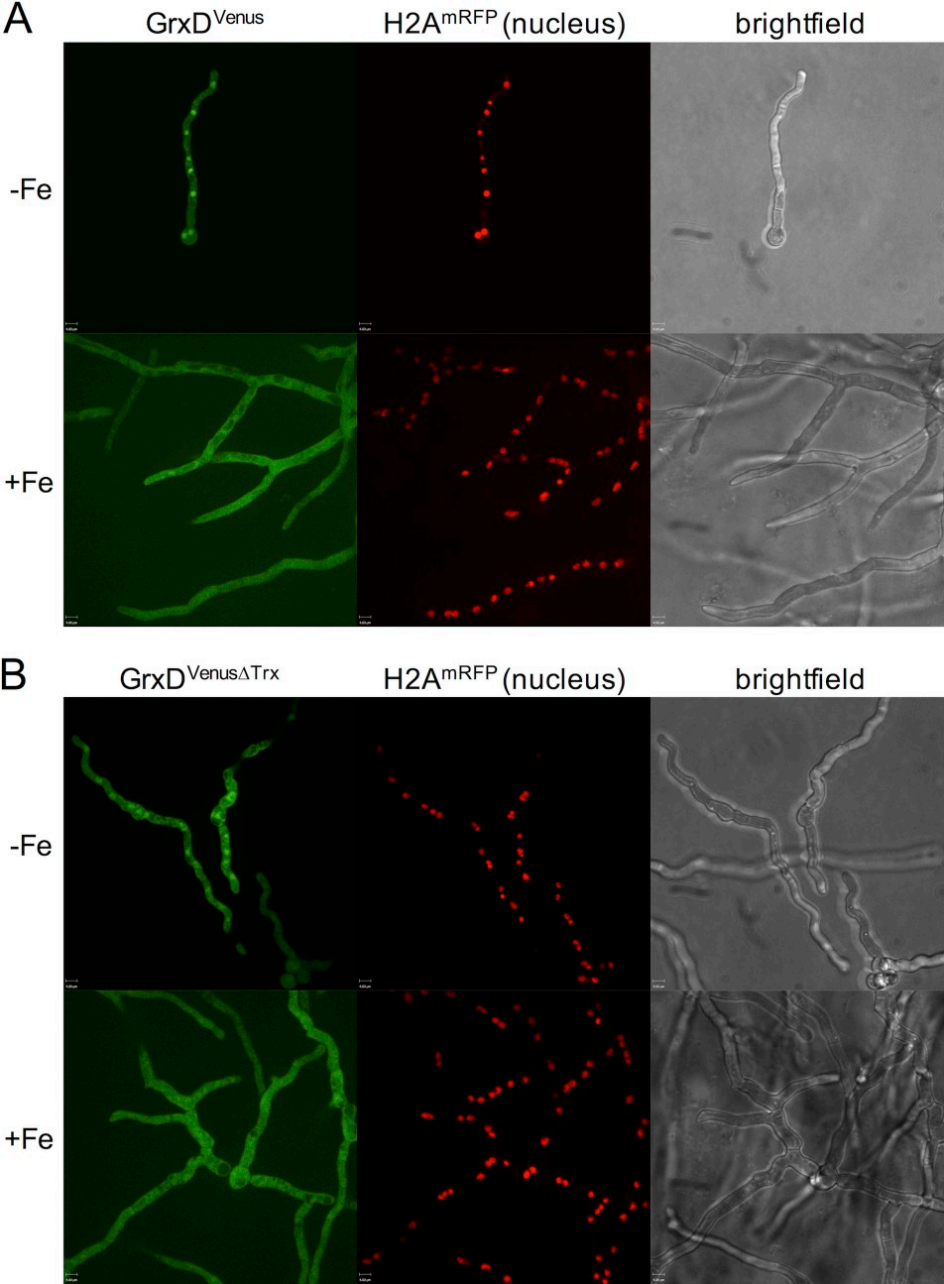
GrxD^Venus^ and GrxD^VenusΔTrx^ are enriched in the nucleus during iron starvation. For fluorescence microscopy, strains (A) *PxylP*:*grxD*^*venus*^/*H2A*^*mRFP*^ and (B) *PxylP*:*grxD*^*venusΔtrx*^/*H2A*^*mRFP*^ were grown for 18h with 0.05% xylose under iron starvation (-Fe) or iron sufficiency (+Fe). The mRFP-tagged histone H2A served to visualize nuclei.

Fluorescence microscopy with these strains revealed that GrxD^Venus^ and GrxD^VenusΔTrx^ displayed predominant nuclear localization during iron starvation but not iron sufficiency (Fig 4). During iron sufficiency, we did not observe organelle-specific accumulation of GrxD^Venus^. The nuclear localization indicates a regulatory role of GrxD at least during iron starvation. Noteworthy, it has been demonstrated previously that HapX also accumulates in the nucleus during iron starvation [3].

### GrxD associates with the iron responsive transcription factors HapX and SreA as well as with components of the cytosolic iron-sulfur protein assembly (CIA) pathway

**Fig 5:**
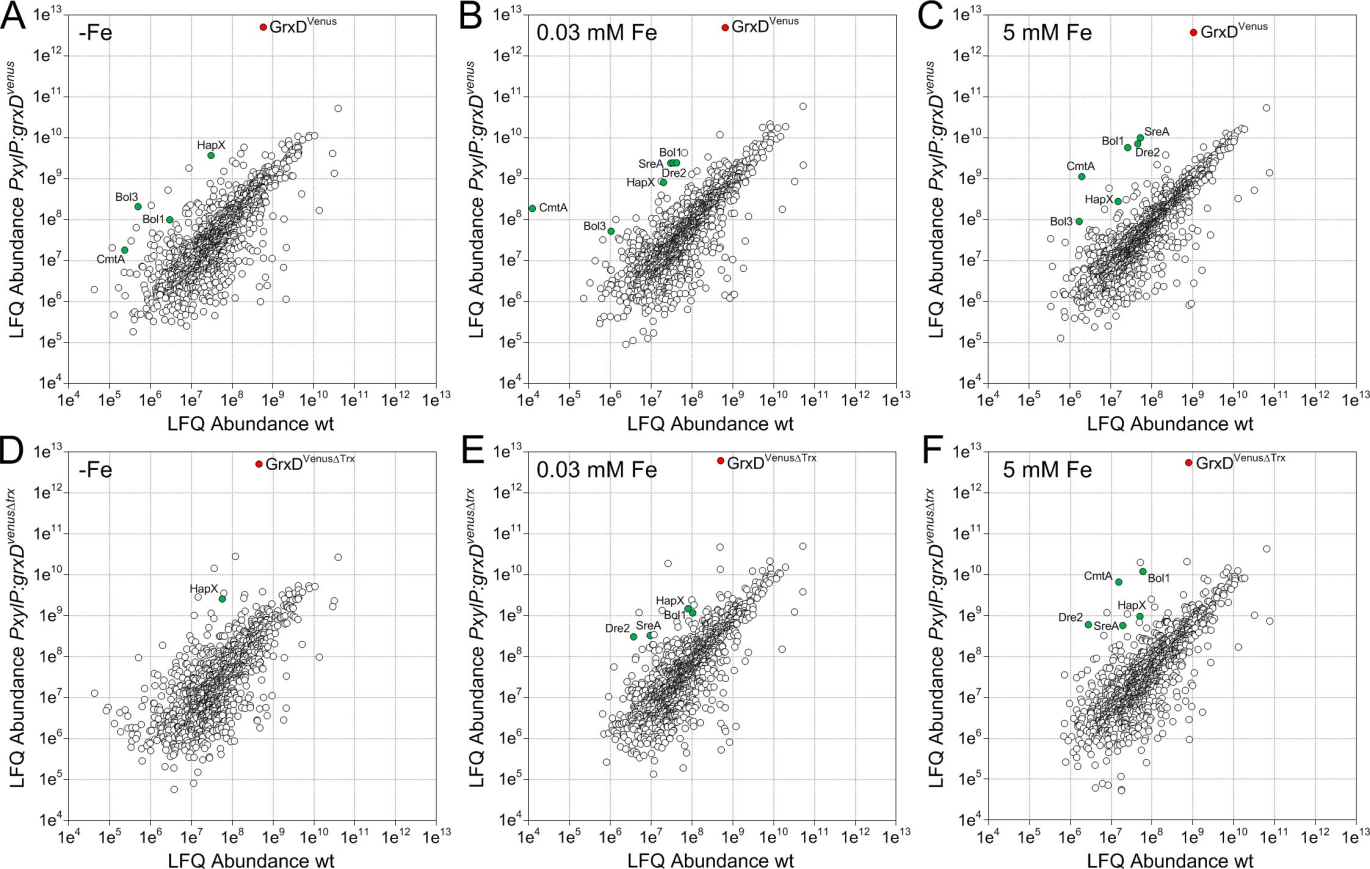
Scatterplot visualization of specifically enriched proteins interacting with Venus tagged GrxD (GrxD^Venus^) and GrxD lacking its Trx domain (GrxD^VenusΔTrx^). Absolute label-free quantification (LFQ) abundances of proteins were determined by nLC-MS/MS analysis after GFP-Trap affinity purification from *A*. *fumigatus PxylP*:*grxD*^*venus*^ (A-C) and *PxylP*:*grxD*^*venus*Δ*trx*^ (D-F) mycelial extracts and plotted against their LFQ abundances in wt GFP-Trap eluates. Each dot represents the mean value of three biological replicates. Mycelia were grown under iron starvation (-Fe), sufficiency (0.03 mM Fe), and excess (5 mM Fe) at 37°C for 22 h. Expression of *grxD*^*venus*^ and *grxD*^*venus*Δ*trx*^ under control of *PxylP* was induced by addition of 1% (w/v) xylose (at -Fe) or 0.1% (w/v) xylose (at 0.03 and 5 mM Fe). Specifically co-purified proteins are indicated by green dots. Bait proteins GrxD^Venus^ and GrxD^VenusΔTrx^ are marked as red dots.

To identify GrxD-interacting proteins, *A*. *fumigatus* strains wt, *PxylP*:*grxD*^*venus*^ and *PxylP*:*grxD*^venusΔtrx^ were cultivated under iron starvation (-Fe), sufficiency (0.03 mM Fe) and excess (5 mM Fe) and the corresponding crude cell extracts were subjected to GFP-Trap affinity purification [24]. Here, wt served as a negative control to distinguish specifically interacting proteins from false positive bound ones. Effective enrichment of GrxD^Venus^ and GrxD^VenusΔTrx^ proteins was validated by SDS-PAGE and silver staining as well as Western blot analysis (S5 Fig). Eluates from three independent biological GFP-Trap replicates were subsequently analyzed by nLC-MS/MS. For visualization of the specific enrichment of GrxD-interacting proteins, label-free quantification (LFQ) abundances of the most enriched proteins identified in *PxylP*:*grxD*^*venus*^ and *PxylP*:*grxD*^venusΔtrx^ GFP-Trap eluates were plotted against their LFQ abundances in wt control eluates (Fig 5 and Tables 1 and S1).

**Table 1:** List of the most enriched proteins by GFP-Trap affinity purification using GrxD^Venus^ and GrxD^VenusΔTrx^ as bait proteins. GFP-Trap eluates from *A*. *fumigatus* wt mycelial extracts were used as control. Proteins with LFQ abundances higher than 5x10^7^ that were enriched more than 4-fold (log2) versus wt controls in at least one iron supply condition are presented. Nbp35 and Mms19 are included, but are enriched less than 4-fold (log2). nd, ortholog not determined.

			GFP-Trap enrichment vs. wt (log2)								
Locus			*PxylP*:*grxD*^*venus*^			*PxylP*:*grxD*^venusΔtrx^			Fungal orthologs		
*A*. *fumigatus*	Name	Function (putative)	-Fe	+Fe	hFe	-Fe	+Fe	hFe	*S*. *cerevisiae*	*S*. *pombe*	*C*. *neoformans*
Afu5g03920	HapX	Iron responsive transcription factors	6.82	5.24	4.11	5.39	4.13	4.14	(Yap5)	(Php4)	HapX
Afu5g11260	SreA		0.52	6.21	7.47	-	5.03	4.80	-	Fep1	Cir1
AFUB_008090	Dre2		2.53	5.78	7.18	-0.58	6.26	7.69	Dre2	Dre2	CNAG_01802
Afu2g15960	Nbp35	CIA machinery	0.31	1.04	3.72	-	-	-	Nbp35	Nbp35	CNAG_02231
Afu8g05370	Mms19		1.80	2.41	1.71	-1.35	-1.51	-0.49	Mms19 (Met18)	Mms19	CNAG_02467
Afu7g01520	Bol1	Mitochondrial Fe-S cluster biogenesis	4.95	6.04	7.68	1.88	3.40	7.56	Bol1	Uvi31	CNAG_04817
Afu6g12490	Bol3	Fe-S cluster transfer to mitochondrial clients	8.60	5.53	5.65	-	-	-	Bol3 (Aim1)	Fra3	CNAG_03927
Afu4g10100	-	(Methionine synthase reductase)	7.62	4.11	2.49	-	-	-	nd	SPAC1783.01	nd
Afu7g02320	-	Unknown	7.60	4.39	5.91	5.03	4.04	3.49	nd	nd	nd
Afu8g04090	CodA	Choline oxidase	7.51	5.51	3.99	7.44	4.78	3.52	nd	nd	nd
Afu3g06450	Pmt1	Protein O-mannosyltransferase	7.03	6.10	4.12	-	-	-	Pmt5, Pmt1	Ogm1	CNAG_06834
Afu4g04318	CmtA	(Metallothionein)	6.15	13.79	9.07	-0.55	1.81	8.65	nd	nd	nd
Afu4g14380	-	(Glutathione S-transferase)	4.80	4.53	1.58	3.93	4.70	2.02	nd	nd	nd
Afu2g11740	-	(ATP-dependent peptidase)	4.64	5.95	3.92	-1.20	-1.06	-1.88	Pim1	Lon1	CNAG_01266
Afu1g04660	-	(Ribosomal protein L15)	4.60	2.46	3.24	5.14	3.05	3.71	Rpl15A	Rpl15	CNAG_01486
Afu1g08840	-	(Guanylate kinase)	4.55	3.65	3.00	3.73	2.69	3.35	Guk1	SPBC1198.05	CNAG_01364
Afu1g09170	-	(Protein kinase activator)	4.55	3.68	4.01	2.56	2.46	1.83	Mob2	Mob2	nd
Afu7g03980	-	(Translation initiation factor eIF3m)	4.50	4.08	3.06	6.34	6.09	5.36	nd	Tif313	CNAG_00509
Afu5g01440	-	(Peroxiredoxin)	4.51	3.32	3.49	4.56	3.30	2.42	Ahp1	Pmp20	CNAG_07032
Afu5g12190	-	(Transcription initiation factor subunit)	4.49	3.62	2.01	5.74	4.54	3.70	Taf14	Tfg3	nd
Afu3g04110	-	(snoRNA binding protein)	4.20	2.52	1.86	0.34	-1.92	-2.42	Enp1	Enp1	CNAG_06948
Afu5g04210	-	(Ubiquinol-cytochrome C reductase complex core protein 2)	4.15	1.68	1.23	2.37	0.88	-0.16	Qcr2	Qcr2	CNAG_05179
Afu2g09100	-	(Polyadenylation factor subunit)	4.13	2.09	-	1.07	0.7	-0.66	Rna15	Rna15	CNAG_04541
Afu3g11610	Nhp6	Non-histone chromosomal protein	4.06	2.18	2.34	1.63	-0.95	0.44	Nhp6A	Nhp6	CNAG_06544
Afu2g11540	-	(Ketoreductase)	4.05	4.58	3.55	3.65	4.43	4.29	Ifa38	Ifa38	CNAG_04488
Afu2g11180	FlbA	Developmental regulator	0.13	4.16	3.02	0.54	4.07	4.63	Sst2	Rgs1	CNAG_00125
Afu7g01010	Adh1	Alcohol dehydrogenase	-1,73	0.41	4.57	-	-2.72	-2.27	nd	Adh1	nd

We identified HapX as one of the most highly enriched proteins by GrxD^Venus^ GFP-Trap under iron limitation (Fig 5A). HapX was also detected in iron sufficient and high-iron conditions (Fig 5B and 5C), however, with lower abundance, most likely due to its low protein level under these conditions [3]. Inversely, SreA was preferentially co-purified under iron sufficiency and excess (Fig 5B and 5C), again reflecting the expression pattern of SreA [1]. These data indicate that GrxD constitutively interacts with HapX irrespective of the cellular iron status and at least under iron sufficiency and iron excess also with SreA; possibly, GrxD interacts also constitutively with SreA—the missing detection of the interaction during iron starvation might be due to the low expression of *sreA* during this condition [1].

In addition, proteins that are part of the cytosolic iron-sulfur protein assembly (CIA) machinery, namely Nbp35 (Afu2g15960), Dre2 (AFUB_008090) and Mms19 (Afu8g05370), were enriched with high abundance under standard and excess iron levels (Fig 5B and 5C). The CIA machinery was investigated extensively in the model organism *S*. *cerevisiae*. These studies showed that the monothiol glutaredoxins Grx3 and Grx4 play an indispensable role for cytosolic iron-sulfur (FeS) cluster biogenesis. An early step in cytosolic [4Fe-4S] cluster assembly involves Nbp35 forming a hetero-tetrameric scaffold complex with Cfd1 on which a [4Fe-4S] cluster is bound transiently [25,26]. Dre2 belongs to the CIA electron transfer complex and is needed for formation of the [4Fe-4S] cluster on Nbp35 [27,28]. Mms19 is part of the CIA targeting complex consisting of Cia1, Cia2 and Mms19, which, together with Nar1 transfers the [4Fe-4S] cluster to target apoproteins [29,30].

The precise site of requirement of monothiol glutaredoxins in the cytosolic FeS protein biogenesis has not been determined yet. In yeast, Grx3/4 is required for FeS cluster assembly on Dre2 and Nar1 [19]. How GrxD is exactly involved in the CIA of *A*. *fumigatus* remains to be elucidated. Nevertheless, these data underline the specificity of the approach.

**Fig 6:**
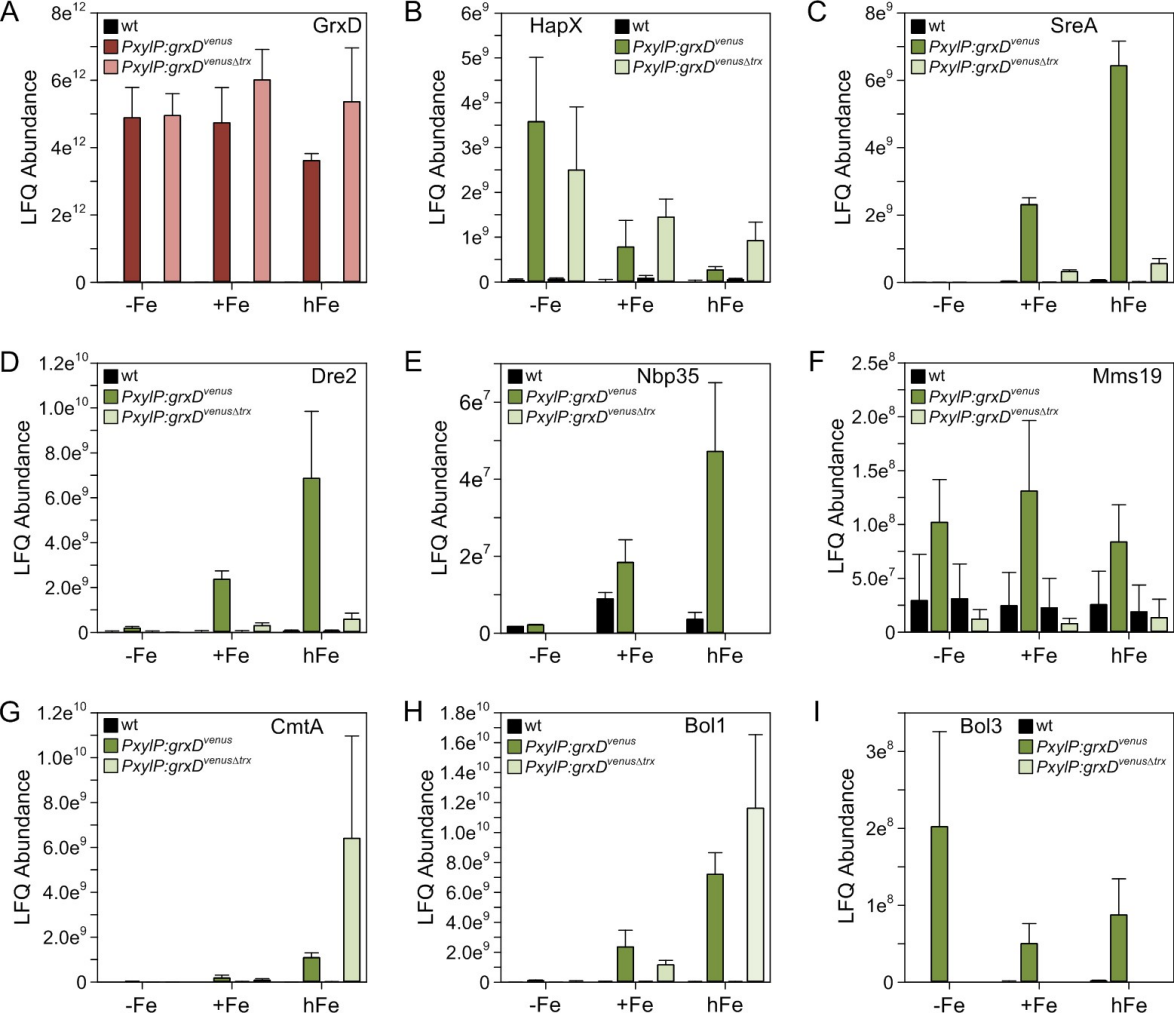
**The GrxD Trx domain is crucial for protein interaction of GrxD with SreA (C), Bol3 (I), and the CIA proteins Dre2 (D), Nbp35 (E) and Mms19 (F), but not for interaction with HapX (B), CmtA (G) and Bol1 (H).** *PxylP*-driven production of GrxD^Venus^ and GrxD^VenusΔTrx^ facilitated trapping of both bait proteins independent from iron supplementation (A).

Grx4 protein interaction studies in *S*. *pombe* demonstrated that the Trx domain is essential for a stable protein interaction with both the iron regulators Fep1 (SreA ortholog) [31] and Php4 (HapX ortholog) [32]. Therefore, we were interested whether the GrxD Trx domain is necessary for all of the detected GrxD protein interactions in *A*. *fumigatus*. To address this topic, we analyzed our quantitative GrxD^Venus^ and GrxD^VenusΔTrx^ GFP-Trap co-purification data for selected interaction partners in detail (Fig 6). The GrxD Trx domain appeared to be dispensable for GrxD-HapX complex formation irrespective of the iron supplementation (Fig 6B). In contrast, the GrxD Trx domain was essential for GrxD-SreA protein interaction (Fig 6C) indicated by a severely decreased SreA LFQ abundance in the absence of the Trx domain. Likewise, GrxD^Venus*Δ*Trx^ pull-down enrichment of the CIA proteins Dre2, Nbp35 and Mms19 was less effective (Fig 6D–6F).

Unexpectedly, we identified the putative copper metallothionein CmtA (encoded by Afu4g04318) as an interaction partner of GrxD, preferably under iron excess conditions (Fig 6G). A recent study regarding *cmtA* regulation and CmtA protein function in *A*. *fumigatus* [33] revealed that *cmtA* expression is not regulated by copper availability and that CmtA is not required for copper detoxification. Consistently, the *cmtA* ortholog in *A*. *nidulans* (AN7011), termed MtlA, was found to be dispensable for copper ion tolerance [34]. Our GFP-Trap pull-down results may suggest that a GrxD-CmtA complex is involved in iron detoxification and/or transport, however this hypothesis has to be verified by future experiments.

Furthermore, our data suggested an interaction of GrxD with two putative BolA family proteins, Bol1 (Afu7g01520) and Bol3 (Afu6g12490). The Trx domain was dispensable for GrxD-Bol1 interaction, but GrxD-Bol3 interaction was dependent on its presence (Fig 6H and 6I). However, both *A*. *fumigatus* proteins contain an N-terminal mitochondrial targeting sequence, suggesting that these proteins are localized in mitochondria. In support, homologs of *A*. *fumigatus* Bol1 and Bol3 from other *Aspergillus* species also contain N-terminal mitochondrial targeting sequences. In agreement, fluorescence microscopy of a strain (*PgpdA*:*bol1*^*venus*^) expressing Bol1 C-terminally tagged with Venus (Bol1^Venus^) suggested that Bol1 is mainly localized in mitochondria (S6 Fig). It has been demonstrated previously that the homologous *S*. *cerevisiae* BolA proteins Bol1 and Bol3 form complexes with mitochondrial Grx5, which lacks a Trx domain [35]. As GrxD is localized in the cytosol and nucleus, the interaction with both mitochondrial Bol1 and Bol3 proteins *in vivo* appears unlikely. One possible explanation for their detected GrxD interaction is the artificial mixture of the proteins when cellular compartments are disrupted during sample preparation. A similar phenomenon has been observed in *S*. *cerevisiae* for interaction of Grx3/4 with Bol1, respectively Bol3 [36], which are both localized in mitochondria [35]. Nevertheless, we can neither exclude that a minor fraction of Bol1 is localized in the cytosol nor that Bol3 is exclusively or partially localized in the cytosol and that GrxD indeed interacts with these BolA-like proteins *in vivo* as described in other organisms [15,31,37,38].

To exemplary confirm GFP trap affinity purification results, we performed co-immunoprecipitation (co-IP) with subsequent Western blot detection (S7 Fig). HapX or SreA, respectively, was immunoprecipitated and purified from *PxylP*:*grxD*^*venus*^ and *PxylP*:*grxD*^*venusΔtrx*^ whole cell lysates using rabbit α-HapX, or rabbit α-SreA antibodies covalently linked to Protein-A-Sepharose. Western blot analysis demonstrated co-IP of GrxD^Venus^ with both HapX and SreA (S7 Fig). These experiments confirmed that GrxD^Venus^ interacts with both HapX and SreA, while truncation of the Trx domain GrxD^VenusΔTrx^ blocks interaction with SreA but not with HapX.

### Co-expression of GrxD and HapX promotes complex formation

**Fig 7:**
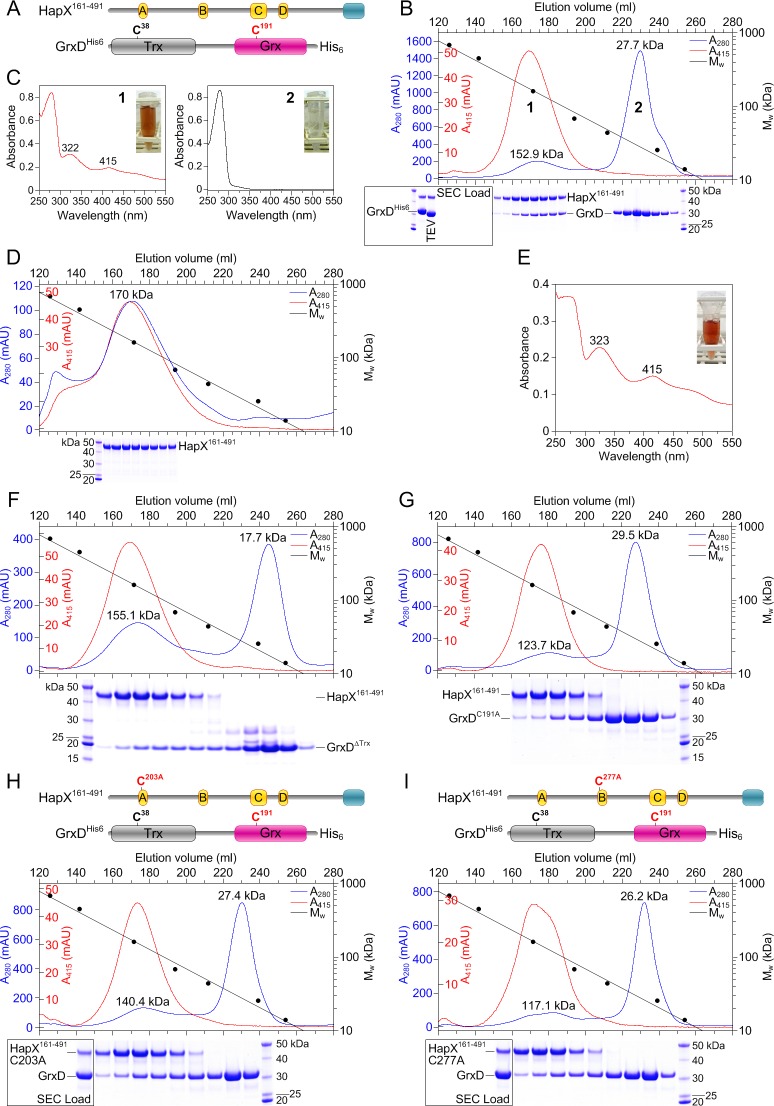
Neither the GrxD Trx domain nor the presence of conserved cysteine residues of GrxD and HapX are required for their *in vitro* protein-protein interaction in *E*. *coli*. (A) Schematic illustration of the co-purified HapX and GrxD polypeptides. The four cysteine-rich regions of HapX^161-491^ are marked in yellow and the C-terminal region, which is essential for low-iron adaptation is shown in turquoise. GrxD thioredoxin (Trx) and glutaredoxin (Grx) domains are marked as grey and pink boxes, respectively. Proteins were enriched from *E*. *coli* cell lysates by Ni-chelate affinity chromatography via the His-tag of GrxD. The His-tag was subsequently removed by TEV protease treatment and proteins were further purified by size exclusion chromatography (SEC). (B) SEC A_280_ and A_415_ elution profiles of GrxD-HapX^161-491^ co-purification and Coomassie-staining of collected protein fractions after separation by SDS-PAGE. Peak 1 contained the GrxD-HapX^161-491^ protein complex with an apparent molecular mass of 152.9 kDa, which corresponds to a complex of two GrxD and two HapX^161-491^ subunits. Peak 2 represented the excess GrxD monomer with an apparent molecular mass of 27.7 kDa. (C) The GrxD-HapX^161-491^ complex (SEC peak1) was reddish-brown colored and the corresponding UV-Vis spectrum displayed absorption maxima at 322 and 415 nm, indicating the incorporation of FeS cluster(s), whereas the GrxD fraction (SEC peak 2) was colorless and lacked absorption maxima at 322 and 415 nm. (D) SEC A_280_ and A_415_ elution profiles and the corresponding (E) UV-Vis spectrum of HapX^161-491^ purified in the absence of GrxD indicating the incorporation of FeS cluster(s) by AfuHapX^161-491^ independent of GrxD. (F) The SEC A_280_ and A_415_ elution profiles of GrxD^ΔTrx^-HapX^161-491^ and (G) GrxD^C191A^-HapX^161-491^ co-purifications indicated that the GrxD Trx domain as well as the GrxD cysteine 191, respectively, are not essential for GrxD-HapX^161-491^ protein interaction. (H) Mutation of HapX^161-491^ C203 to A as well as (I) exchange of C277 to A in the HapX CRR-A and B did not abolish complex formation with GrxD *in vitro*.

For *in vitro* co-purification experiments, *A*. *fumigatus* GrxD was fused with a C-terminal His-tag (GrxD^His6^) and bicistronically co-expressed in *Escherichia coli* with a polypeptide representing the *A*. *fumigatus* HapX C-terminus (HapX^161-491^) that contains all four cysteine-rich regions (CRR; Fig 7A). To investigate the interaction between both proteins, GrxD^His6^ was enriched from crude cell extract via its His-tag using a Ni-Sepharose column. Consequently, co-purification of HapX^161-491^ requires binding to GrxD^His6^. After initial Ni-chelate chromatography, we observed that GrxD^His6^ and HapX^161-491^ were co-enriched (Fig 7B). The GrxD His-tag was subsequently removed by tobacco etch virus (TEV) protease treatment and the GrxD-HapX^161-491^ complex stability was further analyzed by preparative size exclusion chromatography (SEC). Two major peaks appeared during SEC and their apparent molecular masses were estimated based on the elution volumes of protein calibration standards. For peak 1, a molecular mass of 152.9 kDa (Fig 7B) approximately corresponding to a heterotetrameric complex consisting of two HapX^161-491^ and two GrxD subunits (theoretical mass: 130.4 kDa) was calculated. For peak 2, a molecular mass of 27.7 kDa corresponding to a theoretical molecular mass of a GrxD monomer (29.75 kDa) was determined. Additionally, UV-Vis spectra (250–550 nm) were recorded for peak 1 and 2 (Fig 7C). The reddish-brown color of the GrxD-HapX^161-491^ complex (peak 1) as well as the absorption maxima at 322 and 415 nm indicated the incorporation of a [2Fe-2S] ligand, as spectra of [2Fe-2S] proteins are typically more complex than those of [4Fe-4S] proteins, which display only one characteristic peak around 400–420 nm [39]. In contrast, GrxD separated in excess from the GrxD-HapX^161-491^ complex (peak 2) appeared colorless and displayed no absorption at 322 and 415 nm (Fig 7C). We hypothesized that the reddish-brown color of the GrxD-HapX^161-491^ complex is mainly derived from binding of an FeS ligand by HapX^161-491^ CRR. This was supported by SEC purification of HapX^161-491^ in the absence of GrxD, which yielded a reddish-brown colored SEC fraction displaying a UV-Vis spectrum almost identical to that of the GrxD-HapX^161-491^ complex (Fig 7D and 7E). These data strongly indicate that HapX is able to coordinate FeS cluster(s) without GrxD.

To analyze the *in vitro* GrxD-HapX^161-491^ protein-protein interaction in more detail, two GrxD^His6^ mutants were constructed, co-produced with HapX^161-491^ and purified from *E*. *coli* crude cell extracts. Based on the results of the *in vivo* co-purification experiments, the Trx domain was deleted first. Consistent with our *in vivo* data, removal of the GrxD Trx domain had no impact on GrxD^ΔTrx^-HapX^161-491^ protein interaction *in vitro* (Fig 7F). In a second step, GrxD cysteine (C) residue 191 was mutated to alanine (A). GrxD C191 is part of the CGFS active site motif in the Grx domain, which is highly conserved and known to be important for iron sensing through binding of a [2Fe-2S] cluster in *S*. *cerevisiae* [19,40] and *S*. *pombe* [32,41]. In *S*. *pombe*, the CGFS site’s cysteine is required for iron-dependent Grx4-Php4 complex formation [32]. In this study, mutation of the GrxD C191 to A did not influence binding to the HapX^161-491^ CRR in *E*. *coli* (Fig 7G).

HapX harbors four CRR, which might participate in iron sensing. As reported previously [3], CRR-A and B (Fig 7A) are crucial for adaptation to iron excess. In particular, the mutation of C 203 to A in CRR-A or exchange of C277 to A in CRR-B rendered *A*. *fumigatus* more susceptible to iron overload. C277 is part of the CRR-B **C**^**277**^GF**C**SDGTP**C**I**C** motif, which is reminiscent to the **C**GF**C**NDNTT**C**V**C** [2Fe-2S] cluster binding site in *S*. *cerevisiae* Yap5 [12]. To elucidate the impact of both C203 and C277 on GrxD-HapX^161-491^ complex formation, we targeted C203 and C277 by site-directed mutagenesis and replaced them by alanine. Neither HapX^161-491^ C203A exchange nor C277A substitution affected binding of the respective HapX versions to GrxD (Fig 7H and 7I). In summary, we conclude that the Trx domain and residue C191 of GrxD as well as residues C203 and C277 in HapX are not required for *in vitro* complex formation between GrxD and HapX.

### GrxD is required for induction of iron acquisition and repression of iron inducible genes under iron starvation: Functions involving SreA and HapX

As gene deletion was not possible in wt cells, we developed a protein depletion strategy to investigate the effects of GrxD deficiency. We avoided to use strain *ΔgrxD*/*ΔsreA* as it was not possible to measure effects of GrxD deficiency on SreA in this strain and as growth of this mutant was severely impaired. To study the effects of GrxD depletion on iron regulation, we employed *PxylP*:*grxD*^*Δ19*^, which allowed to decrease *grxD* expression to a lethal amount without xylose induction, while growth was fully rescued with a moderate (0.1%) concentration of xylose (see above, Fig 2).

**Fig 8:**
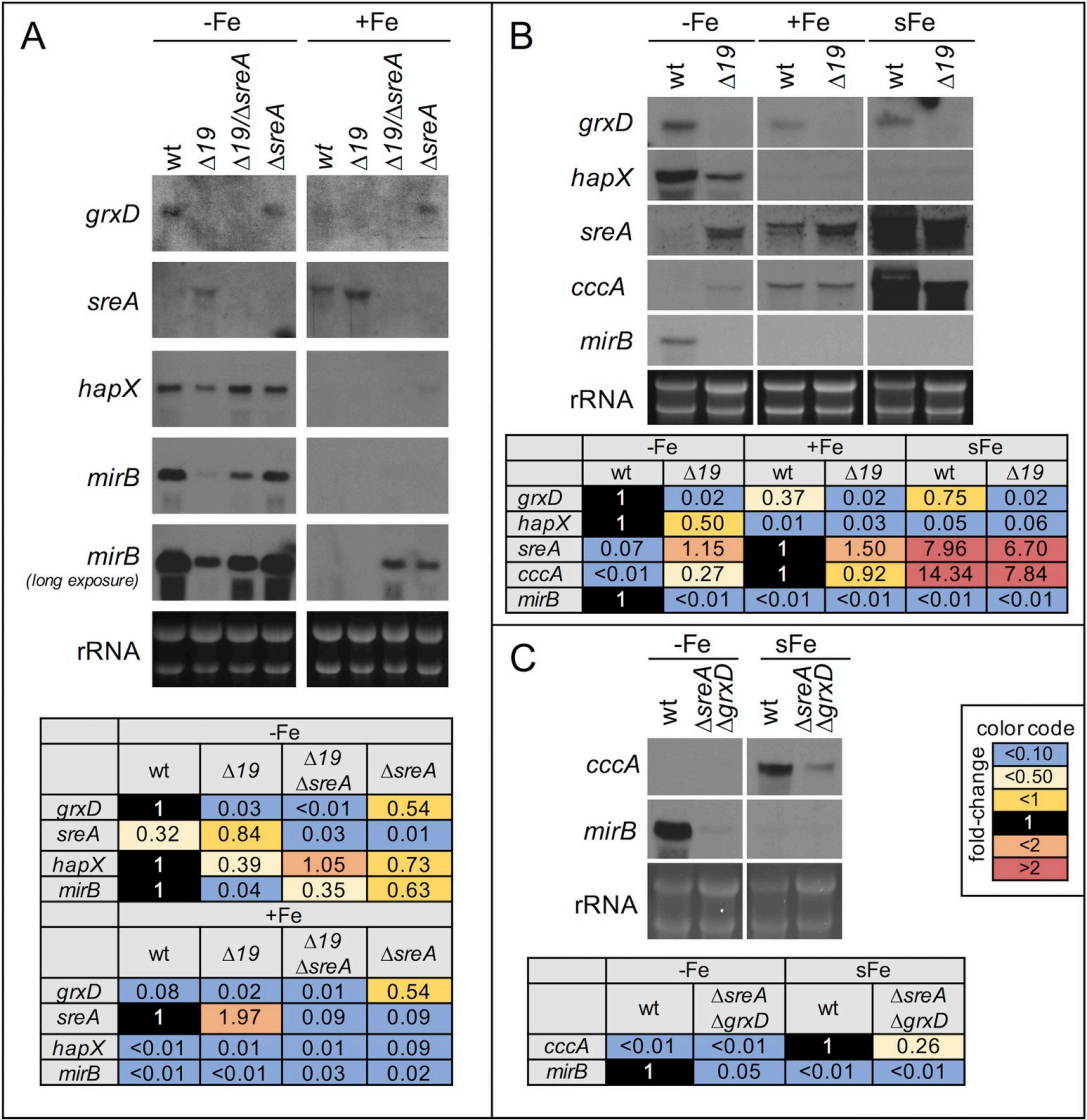
GrxD depletion blocks adaptation to iron starvation, but not adaptation to iron sufficiency/excess. (A) Conidia were germinated with 0.1% xylose (inducing) for 20 h at 25°C. Subsequently spores were washed and cultivated for another 20 h at 37°C without xylose (repressing) to deplete GrxD in *PxylP*:*grxD*^*Δ19*^ strains (indicated as *Δ19*). (B) After growth under iron starvation and depletion of GrxD, 0.03 mM iron was added for 30 min (sFe) to monitor short-term iron response. Similar to wt, iron responsive genes *cccA* and *sreA* were induced in a GrxD-depleted strain, indicating that GrxD is mainly required for sensing of iron starvation. (C) sFe response in Δ*sreA*/Δ*grxD* showed that also gene-deletion of *grxD* does not abolish induction of *cccA*, which indicates a GrxD independent function of HapX. Quantification (Tables) was done as described in Fig 3.

To analyze the effect of GrxD depletion on iron regulation, we performed Northern analysis of iron regulated genes during iron starvation and sufficiency. For GrxD depletion, *PxylP*:*grxD*^*Δ19*^ was grown under inducing conditions for 20 h at 25°C and subsequently grown for another 20h at 37°C without xylose to repress *grxD* expression. This method was used previously to investigate essential genes [23]. During iron starvation, GrxD depletion decreased transcript levels of *hapX* and *mirB*, which were upregulated during iron starvation in wt (Fig 8A and 8B). On the other hand, GrxD depletion increased transcript levels of *sreA* (Fig 8A and 8B) and *cccA* (Fig 8B), which are downregulated during iron starvation in wt. During iron sufficiency, GrxD depletion did not significantly affect transcript levels of these genes. These data emphasize that GrxD is involved in iron regulation and is important for adaptation to iron starvation rather than iron sufficiency.

Repression of *sreA* and *cccA* during iron starvation has previously been shown to depend exclusively on HapX [2,3]. Therefore, the de-repression of these genes found upon GrxD depletion indicates that GrxD is required for signaling iron starvation to HapX. To test whether the effects on *mirB* are linked to SreA or HapX, we also depleted GrxD in strains lacking SreA (strain *PxylP*:*grxD*^*Δ19*^*/ΔsreA*). It has been shown previously that *sreA* is downregulated in wt during iron starvation and lack of SreA results in de-repression of iron-uptake genes (*mirB*, *hapX*) during iron sufficiency [1]. Deletion of *sreA* in *PxylP*:*grxD*^*Δ19*^ increased expression of *mirB* upon GrxD depletion, albeit not to wt level. This indicated that GrxD is required to inactivate the repressing function of SreA under iron starvation. The absence of full induction in GrxD depleted *PxylP*:*grxD*^*Δ19*^*/ΔsreA* compared to the appropriate reference (*ΔsreA*) indicates that GrxD is not only required to inactivate SreA-mediated repression of *mirB*, but also for the induction of *mirB* expression, likely via activation of HapX inducing function.

Interestingly, *grxD* was also de-repressed during iron sufficiency in a SreA deficient strain (Fig 8A), suggesting that SreA is a repressor of *grxD* transcription during iron sufficiency. In agreement, MEME analysis [42] of *grxD* promoter regions of 20 different *Aspergillus* species identified the highly conserved motif 5´-ATCWGATAA-3´ (S8 Fig), which was previously shown to be the consensus motif for DNA-binding by SreA [1]. This regulatory pattern is similar to that in *S*. *pombe*, since *grx4* transcript levels are about 2-fold elevated in iron-starved cells [43], but contrasts the situation in *S*. *cerevisiae* because *grx4* is here under control of Yap5, which activates *grx4* gene expression in iron excess conditions [44].

Previously, HapX was shown to be essential for transcriptional short-term induction of iron-consuming genes [3]. To investigate whether this induction depends on GrxD, we shifted GrxD-depleted cells from iron starvation to iron sufficiency (Fig 8B). Such a shift causes extensive transcriptional rearrangements including repression of iron uptake (mainly via SreA, [1]) and induction of iron-consuming genes (mainly via HapX, [3]). Remarkably, GrxD depletion did not completely block induction of *sreA* and *cccA* in this set-up indicating independence of GrxD.

To prove that this induction is not mediated by remaining GrxD protein levels upon GrxD depletion, Northern blot analysis was performed using strain *ΔgrxD*/*ΔsreA*, which lacks GrxD and SreA. The shift from iron starvation to iron sufficiency still induced *cccA* in this mutant, although the response was decreased compared to wt (Fig 8C). *cccA* is exclusively regulated by HapX [3] and therefore its induction during sFe proves that GrxD is, at least partially, dispensable for HapX function during iron excess. The most likely explanation for the decreased response is the transcriptional downregulation of iron acquisition mechanisms during iron starvation in GrxD-lacking cells (see above), which decreases iron uptake in the iron shift.

In summary, these data indicate that GrxD is required during iron starvation conditions to activate HapX iron starvation function (i.e. repression of iron-consuming genes and induction of iron uptake) and to inactivate SreA function (i.e. repression of iron uptake), but not for iron sensing by HapX under iron excess.

### Cysteine 191 is essential for iron sensing

**Fig 9:**
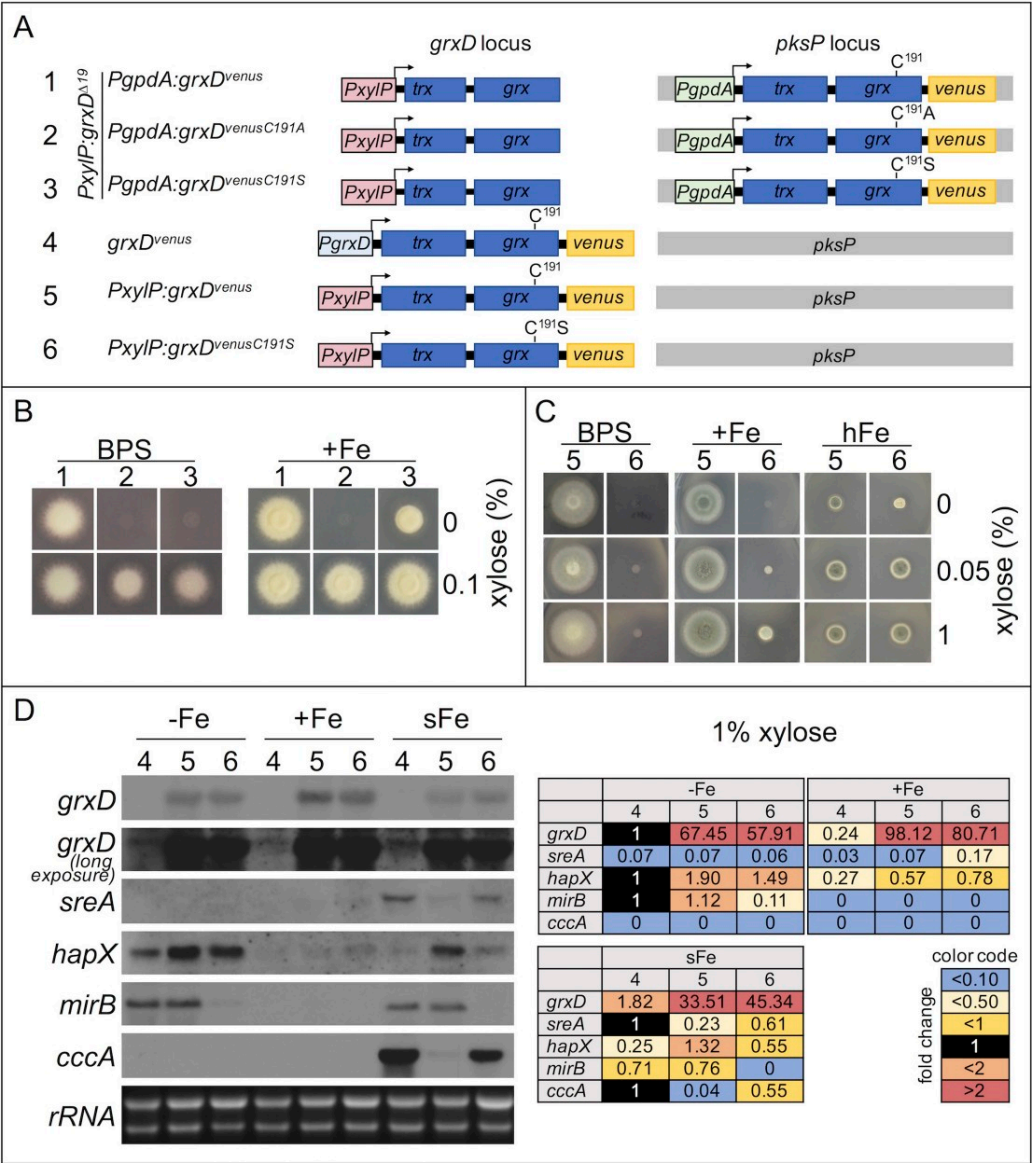
GrxD cysteine residue 191 plays a crucial role in adaptation to iron starvation. (A) Schematic view of strains allowing overexpression (*PgpdA* promoter) of *grxD* variants integrated into the *pksP* locus of strain *PxylP*:*grxD*^*Δ19*^ (1, 2, 3) as well as strains allowing *PgrxD-* (4) and *PxylP-*controlled (5, 6) expression of *grxD* variants from the endogenous locus (B) Growth of strains 1, 2 and 3 under non-inducing conditions (0% xylose) indicating that C191A conversion is lethal, while C191S conversion enables growth under iron sufficiency but not iron starvation. (C) Overexpression of endogenous *grxD* was replaced by *PxylP-*driven *grxD* C191S variant. Overexpression of GrxD carrying a C191S change (strain 5, 1% xylose) rescued growth under high iron supply but only poorly under iron starvation. (D) Northern analysis demonstrating that GrxD and particularly cysteine residue 191 plays a crucial role in transcriptional adaptation to iron starvation. RNA was isolated from *grxD*^*venus*^ (4), *PxylP*:*grxD*^*venus*^ (5) and *PxylP*:*grxD*^*venusC191S*^ (6) after 19h of growth with xylose induction (1%) under iron starvation (-Fe), iron sufficiency (+Fe) and an additional shift for 15 min from iron starvation to iron sufficiency (sFe). Quantification (Tables) was done as described in Fig 3.

FeS clusters in GrxD homologs are coordinated by C191 in the CGFS motif located in the Grx domain (Fig 1). To analyze the function of this cysteine residue in *A*. *fumigatus* iron-regulation, we overexpressed C-terminal *venus*-tagged *grxD*-variants (targeted to the *pksP* locus and expressed under control of the strong constitutive *PgpdA* promoter of glyceraldehyde-3-phosphate dehydrogenase encoding gene) using *PxylP*:*grxD*^*Δ19*^ as recipient strain (Fig 9A). This strategy allowed for growth during induction with xylose regardless of the functionality of the *pksP*-targeted *grxD*-variant due to *grxD*^*Δ19*^ expression of the endogenous *PxylP-*controlled *grxD* gene. Without xylose induction, only the *pksP*-located version is expressed allowing phenotypical characterization of the *pksP*-targeted *grxD* variant.

Overexpression of *grxD*^*C191A*^ was unable to rescue the growth defect caused by lack of GrxD (non-inducing conditions) during iron starvation and iron sufficiency, demonstrating that replacement of cysteine residue 191 by alanine blocks GrxD function (Fig 9B). In contrast, expression of *grxD*^*venusC191S*^ was able to rescue the lack of GrxD during iron sufficiency but not iron starvation (Fig 9B). Similarly, serine can partially compensate for the function of this cysteine residue in the *S*. *cerevisiae* GrxD homolog [19,45]. Endogenous (wt) GrxD protein levels are highest under iron starvation (Fig 3B), indicating a higher GrxD requirement under iron starvation, which might explain the lack of compensation by GrxD^VenusC191S^ under this condition. Alternatively, C191 might be particularly important for adaptation to iron starvation. Interestingly, under xylose-inducing conditions (leading to expression of *grxD*^*Δ19*^) overexpression of *grxD*^*C191A*^ or *grxD*^*C191S*^ decreased growth particularly during iron starvation indicating a dominant negative effect of these GrxD variants.

As overexpression of *grxD*^*venusC191S*^ was partially able to compensate downregulation of *grxD*^*Δ19*^, we generated a mutant strain expressing exclusively *PxylP*-driven *grxD*^*venusC191S*^ (Fig 9A). Indeed, overexpression (xylose-induction) of *grxD*^*venusC191S*^ also enabled growth in this set-up in an iron supply-dependent manner: wt-like (or even better than wt) growth during high iron conditions, decreased growth during iron sufficiency but only poor growth during iron starvation (Fig 9C), as observed above in *PxylP*:*grxD*^*Δ19*^*/PgpdA*:*grxD*^*venusC191S*^ (Fig 9B).

Northern analysis revealed that overexpression of either *grxD*^*venus*^ or *grxD*^*venusC191S*^ increased expression of *hapX* during iron starvation (Fig 9D). As *hapX* expression is mainly regulated by SreA repression, these data indicate that overexpression of either *grxD*^*venus*^ or *grxD*^*venusC191S*^ inactivates SreA. In agreement, GrxD deficiency constitutively activated SreA (Fig 8A). Remarkably, overexpression of *grxD*^*venusC191S*^ but not *grxD*^*venus*^ decreased expression of *mirB* during iron starvation (Fig 9D). This result resembles GrxD deficiency (Fig 8B) and indicates that the residual function of GrxD^VenusC191S^ is not sufficient to maintain the iron-regulatory function under iron starvation. As *mirB* expression requires not only inactivation of SreA (and SreA is highly inactivated as judged by the *hapX* expression) but also induction by HapX, these findings indicate that GrxD^VenusC191S^ fails to activate HapX in contrast to GrxD^Venus^. In contrast to iron starvation, overexpression of *grxD*^*venusC191S*^ or *grxD*^*venus*^ had no significant effect on these genes during iron sufficiency (Fig 9D). Taken together, these data underline the importance of GrxD for sensing of iron starvation.

As shown previously [23] and above (Fig 8B), a short-term shift from iron starvation to iron sufficiency upregulates *sreA* and *cccA*. This response was previously shown to be mediated by HapX [3] and does not require GrxD as shown here (Fig 8B and 8C). Remarkably, however, this regulation was blocked by overexpression of GrxD^Venus^ but not GrxD^VenusC191S^ (Fig 9D). As GrxD dimers are capable of [2Fe-2S] cluster coordination, these data might indicate that GrxD^Venus^ but not GrxD^VenusC191S^ competes with HapX for [2Fe-2S] and thereby blocks activation of the high-iron function of HapX. In agreement, a *grxD*^*venus*^ overexpressing strain displayed severe growth deficiencies at excess iron conditions (Fig 2C). The observed difference between GrxD^Venus^ and GrxD^VenusC191S^ in these experiments is most likely based on the decreased [2Fe-2S] binding affinity of GrxD^VenusC191S^ compared to GrxD^Venus^.

## Discussion

Recently, we have shown that iron sensing in *A*. *fumigatus* depends on a signal from mitochondrial (ISC) but not on cytosolic (CIA) iron-sulfur cluster biosynthesis and on glutathione biosynthesis [23]. Here we demonstrate that *A*. *fumigatus* monothiol glutaredoxin GrxD is required to activate HapX-mediated adaptation to iron starvation as well as for inactivation of SreA during iron starvation. Thereby GrxD acts as sensor for iron starvation, most likely by modulating the signal for iron availability, which is generated by ISC.

GrxD homologs have previously been shown to be involved in iron sensing in the ascomycetous yeast species *S*. *cerevisiae*, *S*. *pombe* and the basidiomycetous yeast species *Cryptococcus neoformans* [15,19,46,47]. Yet, these fungal species and the filamentous ascomycete *A*. *fumigatus* display significant differences with respect to transcriptional iron regulators and the role of the GrxD homologs.

*S*. *cerevisiae* employs two paralogs, Grx3/4, which are essential for growth dependent on the genetic background [19]; in *S*. *pombe*, mutants lacking Grx4 are viable only under microaerophilic conditions [43,46]; in *C*. *neoformans*, deletion of the entire Grx4 gene but not truncation of the Grx domain is lethal [47]. Here we demonstrate that in *A*. *fumigatus* GrxD is essential for growth, whereby the cysteine residue in the Grx domain plays a crucial role, while the Trx domain is dispensable for growth, at least when the Grx domain is overexpressed. As shown for Grx4 in *S*. *cerevisiae* [19], GrxD has most likely also a dual function in *A*. *fumigatus*: a regulatory role in iron sensing as well as in transport of [2Fe-2S] clusters in cellular metabolism. Moreover, Grx3/4 have been suggested to be involved in stress resistance in *S*. *cerevisiae* via affecting actin dynamics and Sir2 glutathionylation [48,49].

In agreement, co-IP approaches revealed physical interaction of GrxD not only with the iron regulators SreA and HapX, but also with CIA components. Likewise, physical interaction of *Arabidopsis thaliana* GrxD homolog GRXS17 and CIA components has been observed previously [50]. Lethality of lack of GrxD might be a synergistic effect of its dual roles. The fact that we found that lack of SreA suppresses the lethal effect of lack of GrxD and that high iron supplementation suppresses the growth defect caused by GrxD downregulation indicates however that the role in iron sensing is the major reason for its essentiality under standard conditions. Our *in vivo* approaches indicated that the Trx domain of GrxD is required for interaction with SreA but not HapX. In agreement, *in vitro* studies with recombinant proteins revealed that neither the Trx domain nor the cysteine residue in CGFS motif in the Grx domain, which is essential for the [2Fe-2S] cluster coordination, are required for physical interaction of GrxD with HapX. Moreover, cysteine residues, which have previously been shown to be essential for *in vivo* function of HapX under high-iron conditions [3], were found to be dispensable for physical interaction of GrxD with HapX.

**Fig 10:**
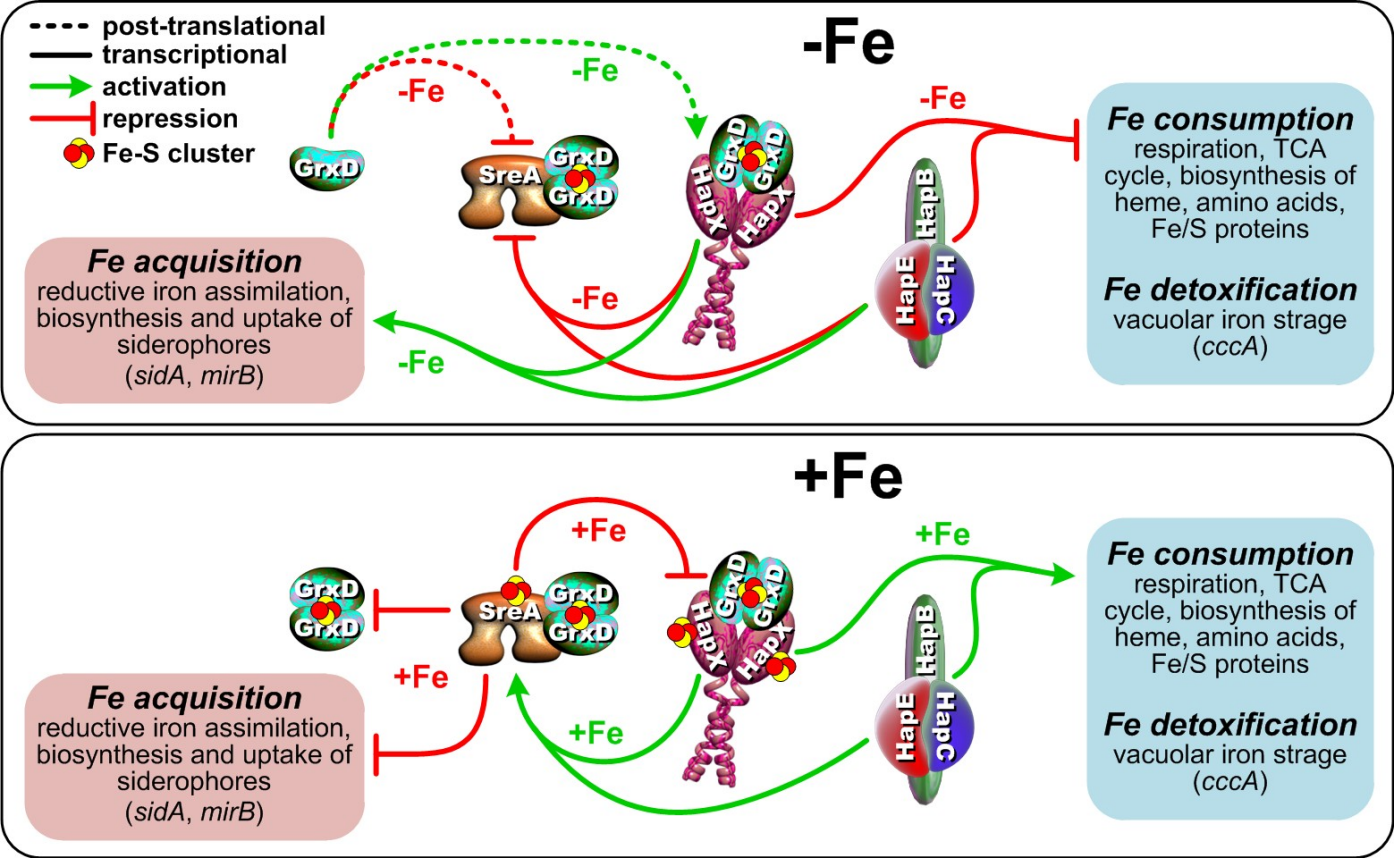
Proposed model for the regulatory function of *A*. *fumigatus* GrxD in iron regulation mediated by SreA and HapX. The two iron-responsive transcription factors SreA and HapX are connected by a negative transcriptional feedback loop. HapX represses *sreA* during iron deprivation (upper panel) and SreA represses expression of *hapX* during iron sufficiency (lower panel). In -Fe conditions, HapX represses genes involved in iron-dependent pathways to save iron and activates iron acquisition. Both HapX functions require the HapB/HapC/HapE complex (CCAAT-binding complex) as DNA-binding scaffold, and as shown in this study the monothiol glutaredoxin GrxD that simultaneously activates the HapX iron-starvation function and disables the repressor function of SreA at the post-translational level. Under iron-replete conditions, SreA represses iron acquisition as well as expression of the GrxD-encoding gene. The latter SreA mode of action represents a novel negative feedback-loop between GrxD and SreA, in which SreA represses its own inhibitor because the repressor function of SreA requires FeS cluster incorporation. Notably, GrxD is dispensable for the HapX iron detoxification function as HapX is able to sense iron levels independent of GrxD by FeS cluster incorporation.

The paralogous *S*. *cerevisiae* transcription factors mediating adaptation to iron starvation, Aft1/2, are conserved exclusively in closely related *Saccharomycotina* and do not display any similarity to HapX or SreA. In *S*. *cerevisiae*, lack of Grx3/4 results in constitutive activation of Aft1/2 irrespective of the iron status. Thus, Grx3/4 is required for inactivation of Aft1/2 during iron sufficiency [15], i.e. sensing of iron sufficiency. The *S*. *cerevisiae* transcription factor mediating adaption to iron excess, Yap5 shows similarities to HapX, but has no function during iron starvation [11]. This indicates that HapX homologs have evolved in a modular manner, whereby *A*. *fumigatus* HapX combines protein modules and respective functions for adaption to iron excess from *S*. *cerevisiae* Yap5 and functions for adaption to iron starvation from *S*. *pombe* Php4 (see below). Similar to Yap5, HapX contains two cysteine-rich regions (CRR), which are crucial for high iron functions [3], whereby one of these contains a perfectly conserved CGFC motif, which was shown to be essential for Yap5 function and [2Fe-2S] cluster coordination [12]. We found in the current study that recombinant HapX displays a reddish-brown color and a UV-Vis spectrum indicative of [2Fe-2S] coordination. Together with our previous observation that activation of the HapX high-iron function depends on ISC but not CIA, our data indicate that HapX senses high iron conditions via [2Fe-2S] coordination similar to Yap5. Remarkably, [2Fe-2S] coordination by Yap5 was shown to be independent of Grx3/4 [12]. Similarly, we also observed that GrxD is dispensable for the activation of the HapX high-iron function in *A*. *fumigatus* (Figs 8B and 8C and 10).

The transcription factors maintaining iron homeostasis in *S*. *pombe* are termed Fep1 and Php4 [13,51]. Fep1 is a homolog of SreA and shares the same function. The HapX homolog Php4 lacks a bZIP-type DNA-binding region but, similar to HapX, interacts with the Php2/Php3/Php5 CBC via its N-terminal CBC-binding domain resulting in repression of iron-consuming pathways under iron starvation [51]. However, in contrast to HapX, Php4 appears to lack a function in activation of iron acquisition during iron starvation and is not involved in adaptation to iron excess. In agreement, the CRR that are conserved and essential for high-iron functions in *S*. *cerevisiae* Yap5 and *A*. *fumigatus* HapX are not conserved in Php4. In *S*. *pombe*, lack of Grx4 caused constitutive activation of the repressing functions of both Php4 and Fep1 [46], i.e. it caused repression of iron acquisition during iron starvation via Fep1 and repression of iron-consuming pathways during iron sufficiency via Php4 and, therefore, deleterious effects during both iron starvation and sufficiency. This finding contrasts the situation in *A*. *fumigatus*, in which lack of GrxD caused regulatory defects only during iron starvation. Thus, GrxD appears to modulate the activity of SreA in *A*. *fumigatus* in a similar way as Grx4 affects Fep1 in *S*. *pombe* (Fig 10). In contrast to Php4 in *S*. *pombe*, however, lack of GrxD did not trigger constitutive HapX iron starvation functions. On the contrary, GrxD depletion impaired HapX mediated adaptation to iron starvation (Fig 10), which indicates significant mechanistic differences in the mode of action of the monothiol glutaredoxin in regulation of *S*. *pombe* Php4 and *A*. *fumigatus* HapX. In *S*. *pombe*, Php4 and Grx4 form a heterodimer, irrespective of the cellular iron status via the Trx domain of Grx4 [32]. During iron sufficiency Php4 and Grx4 are suggested to coordinate a [2Fe-2S] cluster with GSH as additional ligand [16], which causes export from the nucleus to block Php4 activity. In contrast to Php4, HapX appears to coordinate [2Fe-2S] clusters also without GrxD, similar to *S*. *cerevisiae* Yap5 (see above). Unlike *S*. *pombe* Php4, *A*. *fumigatus* HapX also has a function in high-iron conditions and therefore it is unlikely that inactivation of HapX iron-starvation functions (repression of iron-consuming pathways, activation of iron acquisition) involves export of HapX from the nucleus, which could explain evolution of mechanistic differences in modulation of activity of Php4 and HapX.

*C*. *neoformans* employs homologs of *A*. *fumigatus* SreA and HapX, termed Cir1 and HapX, respectively [52,53]. In contrast to *A*. *fumigatus* SreA, however, Cir1 is also involved in adaptation to iron starvation, e.g. activation of iron acquisition. Recently, the GrxD homologue Grx4 was demonstrated to be essential for activation of Cir1 functions via physical interaction, i.e. lack of GrxD phenocopied lack of Cir1 [47]. This differs from the situation in *A*. *fumigatus*, in which lack of GrxD renders SreA constitutively active.

Taken together, the role of GrxD homologs in iron sensing has been demonstrated in different fungal species. In all these species, GrxD homologs display physical interaction with the employed iron regulators. However, these transcription factors show in part significant differences in protein domains and mode of action. These differences are most likely the reason for the different regulatory consequences of lack of GrxD in the analyzed species. Moreover, GrxD homologs show different regulatory patterns in different fungal species. In *S*. *cerevisiae*, expression of the Grx3/4-encoding genes is upregulated during iron sufficiency compared to iron starvation, which is mediated by Yap5 [44]. In contrast, in *S*. *pombe* and *C*. *neoformans*, Grx4 is upregulated during iron starvation compared to iron sufficiency [43,47]. In these species, Grx4 is preferentially located in the nucleus. *C*. *neoformans*, Grx4, however, shows increased nuclear localization under iron starvation compared to iron sufficiency [46,47]. For *A*. *fumigatus* GrxD we found a similar expression and localization pattern as in *C*. *neoformans*. Moreover, we discovered a negative feedback-loop between GrxD and SreA: GrxD is required to repress the function of SreA during iron starvation, while SreA transcriptionally represses expression of the GrxD encoding gene during iron sufficiency (Fig 10).

Iron sensing by *S*. *cerevisiae* Aft1/2 and *S*. *pombe* Fep1 has been shown to involve not only a GrxD homolog but also a cytosolic BolA2-like protein, termed Fra2. In both organisms Fra2 deficiency resembles Grx3/4 or Grx4 deficiency, i.e. a constitutive increase of iron uptake in *S*. *cerevisiae* and constitutive repression of iron uptake in *S*. *pombe* [54,55]. Similar to *S*. *cerevisiae* and *S*. *pombe* [56], the genome of *A*. *fumigatus* and other *Aspergillus* species encodes two BolA-like proteins containing mitochondrial targeting sequences. However, in contrast to *S*. *cerevisiae* and *S*. *pombe*, *Aspergillus spp*. appear to lack a cytosolic BolA2-like protein (although dual localization cannot be excluded) indicating another possible difference in the iron sensing apparatus in these molds.

An intriguing question is of course how GrxD mechanistically modulates the function of SreA and HapX. For *S*. *pombe* it has been suggested that GrxD signals iron starvation to Fep1 by removing iron, not [2Fe-2S], bound by Fep1 [46]. Later on, it was shown that Fep1 coordinates a [2Fe-2S] cluster, not iron, by a highly conserved CRR [57]. Nevertheless, GrxD-mediated removal of [2Fe-2S] clusters bound by SreA and HapX appears to be a conceivable mode of action for signaling iron starvation. Such a model is supported by the fact that overexpression of *grxD* impaired adaptation to iron sufficiency, i.e it blocked short-term induction of *cccA* expression, which depends exclusively on HapX [3]. This effect was not seen when the [2Fe-2S] cluster coordinating cysteine residue in the CGFS motif of GrxD was replaced by a serine residue, which decreases the affinity for the [2Fe-2S] cluster [19]. These data might suggest that in this set-up GrxD competes for [2Fe-2S] clusters with HapX, which impairs iron sensing by HapX. Moreover, this cysteine to serine exchange also impaired transcriptional adaptation to iron starvation, i.e. high-affinity [2Fe-2S] binding by GrxD is crucial for sensing iron starvation. The severe growth defect of downregulation of GrxD in *A*. *fumigatus* is likely a combination of deficiencies in iron sensing and [2Fe-2S] transport. Alternative to GrxD-mediated removal of [2Fe-2S] clusters bound by SreA and HapX, GrxD might signal iron starvation in complexes with HapX and SreA by inducing conformational changes upon [2Fe-2S] cluster coordination. Thus, the cytosolic monothiol glutaredoxin GrxD is involved in iron sensing in *A*. *fumigatus* as shown previously for other fungal species. However, our studies revealed significant differences in the mode of action of GrxD and the consequences of the lack of GrxD in this mold, which underlines a remarkable plasticity in iron sensing in fungi.

The virulence defect of *A*. *fumigatus* mutants lacking siderophore biosynthesis [58–60] or HapX [2], as well as the transcriptional upregulation of iron acquisition pathways [61] in murine infection models indicate that *A*. *fumigatus* faces iron starvation *in vivo*. Moreover, plasma was recently shown to inhibit growth of *A*. *fumigatus* as long as transferrin was not iron saturated, i.e., in the absence of”non-transferrin bound iron” [62]. In line with *A*. *fumigatus* facing iron starvation during growth in plasma we found that GrxD localizes to the nucleus during growth in plasma (S9 Fig) similar to growth during iron starvation in minimal medium (Fig 4). In contrast, supplementation of plasma with high amounts of iron blocked the predominant nuclear localization (S9 Fig) similar to growth under iron sufficiency in minimal medium (Fig 4). Taken together, these data implicate that GrxD plays a role in adaptation to iron starvation during infection. In this regard noteworthy, lack of the Grx domain in the GrxD ortholog renders of *C*. *neoformans* avirulent in a murine infection model [47]. Moreover, the essential role of GrxD for viability of *A*. *fumigatus* underlines the importance of iron metabolism and homeostasis.

## Material and methods

### Strains oligonucleotides and growth conditions

Strains used in this study are listed in S2 Table. Oligonucleotides used in this study are listed in S3 Table. Growth assays were performed in *Aspergillus* minimal medium (1% (w/v) glucose, 20 mM glutamine, salt solution and iron-free trace elements according to [63] and *Aspergillus* complex medium (2% (w/v) glucose, 0.2% (w/v) peptone, 0.1% (w/v) yeast extract, 0.1% (w/v) casamino acids, salt solution and iron-free trace elements according to [63]. Iron (FeSO_4_) was added separately as indicated in the respective figures. However, -Fe, +Fe and sFe stands for iron starvation (no iron), 0.03 mM iron, and shift to 0.03 mM iron after precedent iron starvation, respectively. *PxylP*-driven genes are repressed unless xylose (w/v) is added to the medium, which is indicated in the respective Figures. For solid growth, the medium was solidified with 1.8% (w/v) agarose.

### GrxD protein depletion

In phase one, 10^8^ spores of strains of interest were shaken in 50 ml minimal medium +Fe at 25°C with 0.1% (w/v) xylose (inducing conditions to enable GrxD^Δ19^ production and thereby growth) for 20 h. Germlings were centrifuged and washed once with water to remove iron and xylose before being re-suspended in 100 ml minimal medium containing no xylose. To deplete already produced GrxD^Δ19^ in phase two, growth was continued for 20 h at 37°C. During phase two, the growth conditions were -Fe, +Fe or sFe. Controls were treated the same way.

### Microscopy

For microscopy in minimal medium, strains were grown in well chamber slides (Ibidi) with 2 x 10^4^ spores/well (final concentration 10^5^/ml) for 18h at 37°C with 0.05% (w/v) xylose under iron starvation (-Fe) or iron sufficiency (+Fe). Growth in these chamber slides was hardly sufficient to generate iron starvation after 18 h. To increase iron starvation, -Fe media contained 0.5 mM of the ferrous-iron chelator bathophenanthroline disulfonic acid (BPS). For growth in human blood plasma, spores were inoculated in plasma without or with spiking with 0.1 mM iron to override iron starvation. Spore inoculation and incubation was identical to microscopy with minimal medium. Human plasma was obtained from the bloodbank of Medical University Innsbruck and treated as described previously [62].

Mycelia were examined with a spinning-disc confocal microscopic system (Ultra VIEW VoX; PerkinElmer, Waltham, MA) that was connected to a Zeiss AxioObserver Z1 inverted microscope (Zeiss, Oberkochen, Germany). Images were acquired with Volocity software (PerkinElmer) with a 63x oil immersion objective with a numerical aperture of 1.4. The laser wavelengths used for excitation of Venus and mRFP were 488 and 561 nm, respectively

### Generation of mutant strains

A schematic overview for the generation of all mutant strains is given in S2 Fig.

### *PxylP*:*grxD* and *PxylP*:*grxD*^*venus*^

To simultaneously exchange the endogenous promoter of *grxD* and include a Venus-tag, a plasmid containing *grxD* 5’-region, *hph*, *PxylP*, *grxD* (including 3’-region) and pUC19 backbone was generated. Parts of this plasmid were amplified with primers oKM11-16 and pMMHL15 [23] or *A*. *fumigatus* wt gDNA as template and finally assembled with NEBuilder (New England Biolabs) in pUC19 yielding plasmid pKM1. Subsequently pKM1 was linearized with oKM26 and oKM27 to integrate the *venus*-tag (amplified with oKM28 and oKM29 from phapX^VENUS^-hph [3]) via seamless cloning (NEBuilder; New England Biolabs) yielding pKM1+venus. The insert of pKM1+venus was amplified with primers oKM11 and oKM16 and transformed into a wt recipient strain via homologous recombination. Thereby endogenous *grxD* was exchanged. As two possibilities for homologous recombination at the *grxD* locus were available (S2 Fig), we received two types of transformants, *PxylP*:*grxD* and *PxylP*:*grxD*^*venus*^.

### *PxylP:grxD*^*venusΔtrx*^

Site-directed mutagenesis (Q5 Site-Directed Mutagenesis Kit; New England Biolabs) was performed with pKM1+venus (see above) and primers oMM182 and oMM184 yielding pMMHL43. The insert was amplified with oKM11 and oKM16 and transformed into a wt recipient strain yielding strain *PxylP*:*grxD*^*venusΔtrx*^ via homologous recombination at the *grxD* locus. Thereby endogenous *grxD* was exchanged.

### *PxylP*:*grxD*^*venus*^*/H2AmRFP* and *PxylP*:*grxD*^*venusΔtrx*^*/H2AmRFP*

To integrate mRFP-tagged histone H2A driven by constitutive *gpdA* promoter, a plasmid containing fragment *PgpdA*:*mRFP*:*H2A*, a phleomycin resistance cassette (*ble*), a *pksP* homologous site and pUC19 backbone was generated. Subunits of this plasmid were amplified with primers oMM189-194 and plasmid pME3173 [64], *A*. *fumigatus* wt gDNA or pAN8-1 [65], respectively, as template and finally assembled with NEBuilder (New England Biolabs) in pUC19 yielding plasmid pMMHL44. The plasmid was linearized with *Bam*HI and integrated into the *pksP* locus of recipient strains (*PxylP*:*grxD*^*venus*^ or *PxylP*:*grxD*^*venusΔtrx*^) via homologous recombination at the *pksP* locus. This gene encodes for a polyketide synthase, which is involved in conidial pigmentation [66]. Disruption of *pksP* allows for fast screening of positive integrations, as *ΔpksP* strains produce white conidia.

### ΔsreA

To delete *sreA*, a plasmid containing *sreA* 5’-region, a pyrithiamine resistance cassette (ptrA), *sreA* 3’-region and pUC19 backbone was generated. Subunits of this plasmid were amplified with primers oMM164-169 and *A*. *fumigatus* wt gDNA or pSK275 (syn. pME3024 [67]) as template and finally assembled with NEBuilder (New England Biolabs) in pUC19 yielding plasmid pMMHL38. The insert of pMMHL38 was amplified with oMM164 and oMM169 and transformed into a wt recipient strain. Thereby *sreA* was deleted via homologous recombination.

### *grxD*^*venus*^

*venus*-tagging of *grxD* was performed by employing CRISPR technology as described previously [68]. We used the hygromycin resistance-mediating AMA-plasmid pFC332 and *grxD* targeting sequence AGGCTCCTGCCAGCGCTTGA as protospacer sequence, yielding pMMHL49. A repair template was amplified with oKM15 and oKM16 from pKM1+venus (see above). The repair template and pMMHL49 were together transformed into a wt recipient strain. This procedure caused cleavage at the *grxD* locus by CRISPR and integration of the repair template via homologous recombination. By subsequent growth on non-selective media the CRISPR plasmid was lost yielding *grxD*^*venus*^, a marker-free strain, in which endogenous *grxD* is tagged with *venus* without further manipulation of the *grxD* locus.

### *PxylP*:*grxD*^*Δ19*^ and *PxylP*:*grxD*^*Δ19sup*^

The 5’-region of *grxD* was amplified with primers oAfgrx4-oe1 and oAfgrx4-oe2 and digested with *Avr*II (fragment A). Truncated *grxD* was amplified with primers oAfgrx4-oe4 and oAfgrx4-oe5 and digested with *Nco*I. The *PxylP* sequence was liberated from plasmid pxylP^p^ [69] by digestion with *Not*I and *Nco*I. Both, truncated *grxD* and *PxylP* were ligated via their *Not*I overhang, the fragment was amplified with primers oAfgrx4-oe6 and oAfgrx4-oe7 and digested with *Xba*I (fragment B). The hygromycin resistance cassette was released from plasmid pAN7-1 by digestion with *Xba*I and *Avr*II (fragment C). Fragments A, B and C were ligated via *Avr*II and *Xba*I overhangs. The resulting fragment was amplified with primers oAfgrx4-oe3 and Afgrx4-oe8 and integrated into a wt recipient strain via homologous recombination at the *grxD* locus yielding *PxylP*:*grxD*^*Δ19*^. Thereby endogenous *grxD* was exchanged. As *grxD* is essential (see Results) growth under non-inducing conditions (no xylose) was inhibited. However, streaking out >10^8^ spores on non-inducing agar plates yielded colonies. At least one of these, designated as *PxylP*:*grxD*^*Δ19sup*^, harbored a mutation suppressing the lethal effect caused by *grxD* deficiency.

### *PxylP:grxD*^*venusΔ19*^

A construct containing *grxD* 5’-region, *hph*, *PxylP* and the 19 aa truncated version of *grxD* as 3’-homologous region was amplified from strain *PxylP*:*grxD*^*Δ19*^ gDNA with primers oAfgrx4-1 and oAfgrx4-oe5 and transformed into *grxD*^*venus*^ as recipient strain via homologous recombination.

### *PxylP*:*grxD*^*Δ19*^*/ΔsreA* and *PxylP*:*grxD*^*Δ19*^*/ΔhapX*

To inactivate *sreA* or *hapX* in a *PxylP*:*grxD*^*Δ19*^ background, the knockout constructs were amplified from *ΔsreA* or *ΔhapX* gDNA with primers oMM164 and oMM169 or oAfhapX-1 and oAfhapX-2, respectively, and transformed into a *PxylP*:*grxD*^*Δ19*^ recipient strain via homologous recombination yielding strains *PxylP*:*grxD*^*Δ19*^*/ΔsreA* and *PxylP*:*grxD*^*Δ19*^*/ΔhapX*.

### Heterokaryon rescue and generation of *ΔgrxD/ΔsreA*

To inactivate *grxD*, a plasmid containing *grxD* 5’-region, *hph*, *grxD* 3’-region and pUC19 backbone was generated. Subunits of this plasmid were amplified with primers oMM301-306 and *A*. *fumigatus* wt gDNA or pAN7-1 [70] as template and finally assembled with NEBuilder (New England Biolabs) in pUC19 yielding plasmid pMMHL61. The insert of pMMHL61 was amplified with oMM301 and oMM306 and transformed into wt as recipient strain. This procedure yielded heterokaryotic transformants, containing two different nuclei (*grxD*^*+*^*hph*^*-*^; wt; containing *grxD* but lacking *hph* and *grxD*^*-*^*hph*^*+*^; *ΔgrxD;* lacking *grxD* but containing *hph*) as described in Results. The amplified cassette was also transformed into *ΔsreA* as recipient strain. Thereby *grxD* was deleted via homologous recombination.

### *PxylP*:*grxD*^*Δ19*^*/PgpdA*:*grxD*^*venus*^, *PxylP*:*grxD*^*Δ19*^*/PgpdA*:*grxD*^*venusC191A*^ and *PxylP*:*grxD*^*Δ19*^*/PgpdA*:*grxD*^*venusC191S*^

To rescue *grxD* deficiency, a plasmid was generated containing *pksP* and *PgpdA*:*grxD*:*venus* in backbone *PgpdA-lacZ-trpC*T-pJET1.2 [71]. The *pksP* fragment was amplified with oAf-pksP1-f and oAf-pksP2-r and integrated into the *Hind*III site of *PgpdA-lacZ-trpC*T-pJET1.2 yielding pMMHL6. Subsequently, pMMHL6 was partially amplified with oMM156_HL6fwd and oMM157_HL6rev and assembled with *grxD*:*venus* amplified from pKM1+venus with primers oMM158_grxDfwd and oMM159_venus_rev using NEBuilder (New England Biolabs). The resulting plasmid pMMHL37 was used for site-directed mutagenesis (Q5 Site-Directed Mutagenesis Kit; New England Biolabs) with primers oMM313 and oMM314 or oMM314 and oMM315 to generate pMMHL63 or pMMHL64, respectively. pMMHL37, pMMHL63 and pMMHL64 were linearized with FseI and transformed into *PxylP*:*grxD*^*Δ19*^ as recipient strain to obtain strains *PxylP*:*grxD*^*Δ19*^*/PgpdA*:*grxD*^*venus*^, *PxylP*:*grxD*^*Δ19*^*/PgpdA*:*grxD*^*venusC191A*^ and *PxylP*:*grxD*^*Δ19*^*/PgpdA*:*grxD*^*venusC191S*^ via homologous recombination in locus *pksP*.

### *PxylP:grxD*^*venusC191S*^

To exchange endogenous *grxD* by a *PxylP*-driven *grxD* version in which cysteine 191 is replaced by serine, pKM1+venus was used for site directed mutagenesis (Q5 Site-Directed Mutagenesis Kit; New England Biolabs) with primers oMM313 and oMM314 yielding pMMHL65. The insert was amplified with oKM11 and oKM16 and transformed into a wt recipient strain yielding strain *PxylP*:*grxD*^*venusC191S*^ via homologous recombination in locus *grxD*. Thereby endogenous *grxD* was exchanged.

### *PgpdA:bol1*^*venus*^

To constitutively express *venus* tagged *bol1* from the *pksP* locus, a plasmid was generated consisting of *PgpdA*-driven *bol1* followed by *venus* assembled in pMMHL37 as backbone. Therefore, *bol1* was amplified with primers oMM358 and oMM359 from *A*. *fumigatus* wt gDNA and assembled with linearized pMMHL37 (linearized with primers oMM356 and oMM357) in a NEBuilder (New England Biolabs) reaction yielding the final plasmid pMMHL83. This plasmid was subsequently linearized with FseI and integrated into locus *pksP* via homologous recombination.

### Nucleic acid isolation, Northern analysis, Southern analysis

RNA was isolated using TRI Reagents (Sigma) according to the manufacturer’s manual. 10 μg of RNA was used for electrophoresis on 2.2 M formaldehyde agarose gels and subsequently blotted onto Amersham Hybond-N Membranes (ThermoFisher). Transcripts of interest were detected with DIG-labeled probes amplified by PCR.

DNA was isolated by PCI extraction and isopropanol precipitation. To confirm the gene-specific restriction pattern of the genetic manipulations, DNA was digested with restriction enzymes specific for the respective gene. The resulting restriction fragments were separated on an agarose gel and transferred to Amersham Hybond-N Membranes (ThermoFisher) by capillary blotting with NaOH. Signals for correct integration were detected with DIG-labeled probes amplified by PCR.

### Rabbit polyclonal antisera against HapX and SreA

Rabbits were immunized with polypeptides corresponding to the amino acid residues of HapX^161-491^ and SreA^308-546^. Sequences were PCR-amplified as NdeI-NotI fragments from cDNA and inserted into a pET-21b(+) vector (Novagen) to obtain polypeptides with a C-terminal 6x-His tag. The resulting plasmids were introduced into *E*. *coli* Rosetta BL21 cells (Novagen), designed to enhance the expression of eukaryotic proteins that contain codons rarely used in *E*. *coli*. Expression was induced for 4 h at 37°C with 0.1 mM isopropyl-β-D-thiogalactopyranoside (IPTG). Proteins were purified from cleared lysates by incubation, 2 h at 4°C, with 0.5 ml of Ni-NTA Agarose Resin (Qiagen). Beads were washed repeatedly with phosphate buffer saline (PBS) containing 75 mM imidazole followed by PBS with 100 mM imidazole before proteins were eluted with 500 mM imidazole. Imidazole was removed by extensive dialysis against PBS. Protein material was lyophilized and used to immunize rabbits (by Davids Biotechnologie GmbH, Regensburg, Germany). The specificity of the obtained antibodies was tested by Western analysis (S10 Fig).

### Western analysis (HapX, SreA, GrxD^Venus^, GrxD^VenusΔ19^, GrxD^VenusΔTrx^)

Proteins were extracted using a reported procedure [72] involving solubilization from lyophilized mycelial biomass with NaOH, followed by their precipitation with trichloroacetic acid (TCA). Aliquots were resolved in 10–12% (w/v) SDS-polycrylamide gels and transferred to nitrocellulose membranes. Western blots were reacted with rabbit α-HapX or rabbit α-SreA antiserum (1:20,000), mouse α-GFP antibody (1:10,000; Roche, 11814460001) mouse α‐Tub antibody (1:10,000; Sigma, T6199) as primary antibodies and with peroxidase coupled antibodies as secondary antibodies (1:10,000; anti-Rabbit; Sigma, A1949 or 1:10,000; anti-Mouse; Sigma, A4416). Proteins were detected using Amersham Biosciences ECL.

### Co-IP assays

Covalent coupling of rabbit α-HapX respectively rabbit α-SreA antibodies (antiserum) to Protein-A-Sepharose (GE Healthcare) was performed according to [73]. For the negative control IgGs contained in preserum were covalently linked to Protein-A-Sepharose. In short: 1 ml of Protein-A-Sepharose slurry (50%) was mixed with 0.5 ml (anti)serum and treated with 20 mM dimethylpimelidate in 0.2 M Na-tetraborate. The reaction was stopped with 0.2 M ethanolamine. Immunoprecipitation assays were performed according to [74]. Mycelia were grown for 16 h in minimal medium containing 0.1% xylose and no iron supplementation for HapX immunoprecipitation, or 0.03 mM iron for SreA immunoprecipitation. For protein extracts, 40 mg of mycelia were grinded and dissolved in 1 ml protein extraction buffer containing 20 mM Tris-HCl pH 8, 110 mM KCl, 10% (v/v) glycerol, 0.1% (v/v) Triton X-100, 1μl BitNuclease (Biotool) and protease inhibitor (cOmplete ULTRA EDTA-free, Roche). Extracts were mixed with 50 μl of covalently linked rabbit α-HapX, rabbit α-SreA or rabbit preserum beads and incubated for 3h at 4°C in a rotating wheel. Subsequently the beads were washed three times (10 min at 4°C in a rotating wheel) with chilled protein extraction buffer and increasing salt concentrations (110 mM, 500 mM and 750 mM KCl). Bound proteins were eluted in 40 μl of Laemmli sample buffer at 95°C. Twenty microliters of aliquots were resolved in 10% SDS-polyacrylamide gels and transferred to nitrocellulose for Venus detection. Venus tagged GrxD or GrxD^ΔTrx^ was detected with mouse α-GFP (1:10,000; Roche, 11814460001) and α‐Tubulin was detected with mouse α‐Tub (1:10,000; Sigma, T6199) as primary antibody and with a peroxidase-coupled secondary antibody (1:10,000; anti-Mouse IgG; Sigma, A4416). HapX respectively SreA were detected with rabbit α-HapX or rabbit α-SreA antisera (1:20,000). To avoid the detection of rabbit IgGs, which were used for the co-IP, a conformation specific anti-Rabbit IgG antibody (1:1000; Cell Signaling Technology, L27A9) was used in combination with a peroxidase-coupled anti-Mouse IgG secondary antibody (1:10,000; Sigma, A4416). For the detection Amersham Biosciences ECL was used.

### RACE

3’ RACE was performed using FirstChoice RLM-RACE Kit (ThermoFisher). Total RNA from *PxylP*:*grxD*^*Δ19sup*^ was reverse transcribed with the oligo-dT containing primer 3' RACE Adapter. The resulting cDNA was used for Touchdown PCR with *sreA* (5’-UTR)-specific forward primer oKM31 and adapter-specific reverse primer 3’ RACE Outer Primer. To increase specificity, the resulting PCR product(s) were amplified in a second PCR with nested primers oKM30 and 3’ RACE Inner Primer. This procedure yielded a fragment (~900bp) which was isolated and sequenced (S3 Fig).

### GFP-Trap immunoprecipitation of GrxD^Venus^ and GrxD^VenusΔTrx^ fusion proteins

*A*. *fumigatus* mycelia were harvested in Stop buffer [75] at 4°C after growth for 22 h and freeze-dried. Protein extraction was performed according to a modified procedure from [75] using HK buffer for total protein extraction. All steps were carried out at 4°C in the cold room. In short, 100 mg of mycelium powder was dissolved in 1 ml HK buffer, centrifuged twice at 20,187 x *g* for 15 min and 500 μl of the supernatant was incubated with GFP-Trap agarose beads (ChromoTek) for 1 h. The beads were washed twice in HK buffer without IGPAL, twice in wash buffer (25 mM Tris/HCl pH 7.5, 500 mM NaCl, 5 mM EDTA and 15 mM EGTA) and once in ultrapure water. Proteins were eluted in 10% (v/v) acetonitrile and 5% (v/v) acetic acid and used for nLC-MS/MS measurement, Western blot detection and silver staining.

### nLC-MS/MS measurement

#### In-solution digest

Dried GFP-Trap eluates were solubilized in 50 μl 50 mM NH_4_HCO_3_ in 50:50 (v/v) trifluoroethanol (TFE)/water. After heat denaturation (90°C, 10 min) the proteins were reduced for 1 h at 55°C by addition of TCEP (tris(2-carboxyethyl)phosphine) at a final concentration of 8 mM. Further carbamidomethylation was performed for 45 min at 32°C in 15 mM chloroacetamide. Subsequently the samples were evaporated in a vacuum concentrator (Eppendorf) to a residual volume of approximately 5 μl. Finally, the volume was set to 30 μl with 50 mM NH_4_HCO_3_ and proteins were digested overnight (18 h, 37°C) with a Trypsin/LysC mixture (Promega) at a protein to protease ratio of 25:1. Peptides were dried in vacuum concentrator and re-solubilized in 20 μl of 0.05% TFA in H_2_O/acetonitrile 98/2 (v/v) and filtered through spin filters. The filtrate was transferred to HPLC vials and injected into the LC-MS/MS instrument. Each sample was measured in triplicate (3 analytical replicates).

### LC-MS/MS analysis

LC-MS/MS analysis was carried out on an Ultimate 3000 nano (n) RSLC system coupled to a QExactive Plus mass spectrometer (both Thermo Fisher Scientific, Waltham, MA, USA). Peptides were trapped for 5 min on an Acclaim Pep Map 100 column (2 cm x 75 μm, 3 μm) at 5 μl/min followed by gradient elution separation on an Acclaim Pep Map RSLC column (50 cm x 75 μm, 2μm). Eluent A (0.1% (v/v) formic acid in water) was mixed with eluent B (0.1% (v/v) formic acid in 90/10 acetonitrile/water) as follows: 0 min at 4% B, 6 min at 6% B, 14 min at 10% B, 20 min at 14% B, 35 min at 20% B, 42 min at 26% B, 46 min at 32% B, 52 min at 42% B, 55 min at 50% B, 58min at 65% B, 60–64.9 min at 96% B, 65–90 min at 4% B. Positively charged ions were generated at 2.2 kV using a stainless steel emitter and a Nanospray Flex Ion Source (Thermo Fisher Scientific). The QExactive Plus was operated in Full MS / data-dependent MS2 (Top10) mode. Precursor ions were monitored at m/z 300–1500 at a resolution of 70,000 FWHM (full width at half maximum) using a maximum injection time (ITmax) of 120 ms and an AGC (automatic gain control) target of 1e6. Precursor ions with a charge state of z = 2–5 were filtered at an isolation width of m/z 1.6 amu for HCD fragmentation at 30% normalized collision energy (NCE). MS2 ions were scanned at 17,500 FWHM (ITmax = 120 ms, AGC = 2e5). Dynamic exclusion of precursor ions was set to 20 s. The LC-MS/MS instrument was controlled by QExactive Plus Tune 2.9 and Xcalibur 3.0 with DCMS Link.

### Protein database search

Tandem mass spectra were searched against the *Aspergillus* Genome Database (AspGD) of *Aspergillus fumigatus* Af293 (http://www.aspergillusgenome.org/download/sequence/A_fumigatus_ Af293/current/A_fumigatus_Af293_current_orf_trans_all.fasta.gz; 2018/09/18) and the protein sequence of Dre2 (AFUB_008090; the Dre2 ortholog is not present in the Af293 gene annotation) as well as further modified protein sequences (e.g. Venus-tag) using Proteome Discoverer (PD) 2.2 (Thermo) and the algorithms of Sequest HT (version of PD2.2) and MS Amanda 2.0. Two missed cleavages were allowed for the tryptic digestion. The precursor mass tolerance was set to 10 ppm and the fragment mass tolerance was set to 0.02 Da. Modifications were defined as dynamic oxidation of Met, acetylation of Ser, phosphorylation of Ser, Thr, and Tyr and ubiquitination (GG) of Lys as well as static Cys carbamidomethylation. At least two peptides per protein and a strict false discovery rate (FDR) < 1% were required for positive protein hits. The Percolator node of PD2.2 and a reverse decoy database was used for q-value validation of spectral matches. Only rank 1 proteins and peptides of the top scored proteins were counted. The Minora algorithm of PD2.2 was applied for relative label-free quantification. GFP-Trap eluates from wt *A*. *fumigatus* mycelial extracts were used for quantification of nonspecifically co-purified proteins.

### Silver staining and Western blot detection of proteins after GFP-Trap

Proteins were separated by SDS-PAGE using NuPAGE 4–12% (w/v) Bis-Tris gradient gels (Invitrogen). Silver staining was performed using the SilverQuest Silver Staining Kit (Invitrogen) according to the manufacturer’s protocol. For Western detection, proteins were transferred onto a PVDF membrane using the iBlot 2 dry blotting system (Invitrogen). The membrane was blocked in 3% (w/v) bovine serum albumin (BSA) dissolved in 1x PBST (137 mM NaCl, 2.7 mM KCl, 10 mM Na_2_HPO_4_, 2 mM KH_2_PO_4_, 0.05% (v/v) Tween 20). As primary antibody rabbit α-GFP (abcam, ab290) was used, followed by secondary antibody HRP-conjugated anti-Rabbit IgG (ICL) incubation. The membrane was developed using the 1-Step Ultra TMB-Blotting chromogenic substrate (Thermo Scientific).

### Expression and purification of recombinant GrxD and HapX^161-491^ proteins from *E*. *coli*

For individual expression and protein purification, synthetic genes coding for full-length GrxD and HapX amino acids 161–491 (cysteine-rich C-terminus) were cloned into the *Nde*I and *Bam*HI sites of the pET-MCN vector pnEA/vH [76] producing C-terminally His_6_-tagged GrxD (pnEA/vH-GrxD) and HapX^161-491^ (pnEA/vH-HapX161-491) fused to a TEV cleavage site. For co-expression, the synthetic gene coding for HapX^161-491^ was initially cloned into the *Nde*I and *Bam*HI sites of the pET-MCN vector pnCS producing untagged HapX^161-491^ (pnCS-HapX161-491). Subsequently, the *Bgl*II/*Xba*I fragment from pnCS-HapX161-491 was subcloned into the *Bgl*II and *Spe*I sites of pnEA/vH-GrxD generating a bicistronic expression cassette. Site-directed mutagenesis was performed with the QuikChange II site-directed mutagenesis kit (Agilent) according to the manufacturer’s protocol. Primers used for mutagenesis are listed in S3 Table.

*E*. *coli* BL21(DE3) cells (New England Biolabs) were transformed with the respective plasmid for autoinduction in Overnight Express Instant TB medium (Novagen). Wet biomass was harvested by centrifugation (10,543 x *g*) and the cell paste was stored at -80°C. Frozen bacterial cells were resuspended in lysis buffer (50 mM HEPES pH 8.0, 300 mM NaCl, 2 mM glutathione, 10 mM imidazole, 1 mM AEBSF) and disrupted at 1000 bar using a high-pressure homogenizer (Avestin Emulsiflex C5). Cell debris were removed by centrifugation (48,384 x *g*), the pH was adjusted to 8.0 and the supernatant clarified by filtration through a 1.2 μm membrane. His_6_-tagged proteins were then purified by Ni-chelate affinity chromatography using a 20 ml Ni-Sepharose FF column (GE Healthcare) and proteins were eluted with 500 mM imidazole. Fractions containing either HapX^161-491^-His_6_ or the HapX^161-491^/GrxD-His_6_ complex were digested with TEV protease for 4 h at room temperature and loaded onto a Superdex 200 prep grade 26/60 size exclusion chromatography column (GE Healthcare) that was equilibrated with 25 mM HEPES pH 7.5, 150 mM NaCl, 2 mM glutathione. UV-Vis absorption spectra were recorded in the range from 250 to 550 nm with a JASCO V-630 spectrophotometer.

## Supporting information

S1 Fig Phylogenetic conservation of GrxD. (A) Alignment of fungal and human GrxD homologs from *A*. *fumigatus* (Afu), *Aspergillus nidulans* (AN), *Neurospora crassa* (Nc), *Candida albicans* (Ca), *S*. *cerevisiae* (Sc), *Cryptococcus neoformans* (Cn), *Ustilago maydis* (Um), *S*. *pombe* (Sp), and *Homo sapiens* (Hs). The Trx-like and Grx domains of AfuGrxD are underlined. Identical residues are marked in yellow, residues conserved in 50% of the sequences are shaded in light blue and blocks of similar residues are marked in green. (B) Phylogenetic tree and (C) Identity table of the aligned amino acid sequences. Numbers in parentheses display the calculated distance values between the sequences. The multiple alignment was performed with AlignX (Vector NTI Advance 11).
(TIF)

S2 Fig Schemes of genetic manipulations of *A*. *fumigatus*. Recipient strains, genetic loci and transformation constructs employed are shown at the left; resulting strains and genetic loci are shown at the right.
(TIFF)

S3 Fig Chromosomal rearrangement within the *sreA* coding region suppresses lethality of lack of *grxD*. (A) Northern analysis of *hapX* and *mirB* in wt, *PxylP*:*grxD*^*Δ19*^, and *PxylP*:*grxD*^*Δ19sup*^ strains under iron starvation (-Fe), iron sufficiency (+Fe) and high iron conditions (hFe) under 0.1% xylose inducing conditions. The additional *sreA* transcript in *PxylP*:*grxD*^*Δ19sup*^ is indicated by a red arrow (B) PCR-amplification analysis demonstrating a recombination in the genomic *sreA* locus: agarose gel electrophoresis, strategy for PCR-amplification of the *sreA* locus and primers employed. The failing PCR amplification of fragments 3, 4, 5, and 9 (entire locus) from genomic DNA of strain *PxylP*:*grxD*^*Δ19sup*^ (mt) compared to wt indicated a breakpoint in exon 1 or 2. (C) Sequence analysis of the amplicon obtained by 3´-RACE from strain *PxylP*:*grxD*^*Δ19sup*^, using *sreA* specific primers located in the *sreA* 5´-UTR, revealed a chimeric mRNA containing the 5´-end of the *sreA* transcript and the 3´-end of the transcript encoded by Afu5g14865. (D) Alignment of wt SreA and the deduced amino acid sequence of the chimeric cDNA obtained by 3´-RACE (mt). This analysis revealed chromosomal recombination within the second GATA-type zinc finger (GTZ; boxed in blue)-coding region of SreA, which caused SreA inactivation. Identical amino acids are indicated by asterisks; differences in the deduced chimeric amino acid sequence are shown in red; CRR (cysteine-rich region) is boxed in yellow. (E) Scheme of the chromosomal rearrangement in *PxylP*:*grxD*^*Δ19sup*^ resulting in inactivation of SreA. (F) PCR-amplification analysis (agarose gel electrophoresis) of the *sreA* locus of *PxylP*:*grxD*^*Δ19sup*^ (mt) compared to wt proving the inversion.
(TIFF)

S4 Fig Strain *PxylP*:*grxD*^*venusΔ19*^ phenocopies strain *PxylP*:*grxD*^*Δ19*^. Strains were grown for 48 h at 37°C in minimal medium under non-inducing (0% xylose) and inducing (0.1% xylose) conditions with iron starvation (-Fe), iron sufficiency (+Fe) and iron excess (hFe), respectively, as described in Fig 2.
(TIFF)

S5 Fig Silver staining and α-GFP Western blot analysis of GFP-Trap affinity purification eluates from *A*. *fumigatus* wt, *PxylP*:*grxD*^*venus*^ and *PxylP*:*grxD*^*venusΔtrx*^ crude cell extracts. (TIF)

S6 Fig Bol1^Venus^ localizes to mitochondria. For fluorescent microscopy, strain *PgpdA*:*bol1*^*venus*^ was grown for 18 h in minimal medium. To visualize mitochondria, the mitochondria specific dye tetramethylrhodamine (TMRM) was used.
(TIFF)

S7 Fig GrxD^Venus^ interacts with both HapX and SreA; truncation of the Trx domain impairs interaction with SreA but not with HapX. HapX and SreA, respectively, were immunoprecipitated with indicated antisera (IgGs covalently linked to Protein-A-Sepharose) in cell free protein extracts obtained from Venus-tagged GrxD or GrxD^ΔTrx^ producing strains *PxylP*:*grxD*^*venus*^ or *PxylP*:*grxD*^*venusΔtrx*^, respectively, grown for 16 h in 0.1% xylose containing minimal medium without iron supplementation for HapX or 0.03 mM iron supplementation for SreA. Immunoprecipitates (IP) were analyzed for Co-IP of GrxD^Venus^ or GrxD^VenusΔTrx^ by immunoblot analysis (IB) with a mouse α-GFP antibody. Successful precipitation of HapX respectively SreA was analyzed by IB analysis with rabbit α-HapX or rabbit α-SreA antisera. HapX levels in the input were below the detection limit. Tubulin was used as a loading control.
(TIFF)

S8 Fig The *grxD* promoter contains a highly conserved putative SreA binding motif. MEME motif 1 in *PgrxD* of 20 *Aspergillus* spp. (SreA target motif 5´-ATCWGATAA-3´). For promoter analysis, the complete 5´ intergenic non-coding *grxD* regions were selected. Putative transcription factor motifs were identified using the MEME motif discovery tool provided by the MEME suite platform. The following parameters were used: motif width 6–16 bp; zero or one occurrence per sequence. In the first ranked motif 20 sites were counted with an E-value of 1.0e-067.
(TIF)

S9 Fig GrxD^Venus^ is enriched in the nucleus during growth in plasma without iron supplementation. For fluorescent microscopy, strain *PxylP*:*grxD*^*venus*^/*H2A*^*mRFP*^ was grown for 18h with 0.05% xylose under iron starvation (-Fe) or iron sufficiency (+Fe). The mRFP-tagged histone H2A served to visualize nuclei.
(TIFF)

S10 Fig Rabbit polyclonal antisera against HapX and SreA. (A) Coomassie-stained gels of HapX^161-491^-(HIS)_6X_ and SreA^308-546^-(HIS)_6X_ polypeptides after purification (see materials and methods). Lanes 3, 4 and 5 of each gel show the amount of protein in 2.5, 5 and 10 μl. 1 and 5 μg of BSA were loaded as controls in lanes 1 and 2, respectively. (B) Western blot analysis with rabbit α-HapX or rabbit α-SreA antisera, and their respective pre-sera as negative controls. Strains were grown in -Fe (for α-HapX blot) and +Fe (for α-SreA blot) minimal medium for 20 h at 37°C. α-Tubulin was used as loading control.
(TIFF)

S1 Table Absolute label-free quantification (LFQ) abundances of proteins identified by nLC-MS/MS analysis after GFP-Trap affinity purification from *A*. *fumigatus* wild type, *PxylP*:*grxD*^*venus*^ and *PxylP*:*grxD*^venusΔtrx^ mycelial extracts. (XLSX)

S2 Table Strains used in this study. For Stains: Δ indicates loss of gene-function, exchanged promoters are indicated with *P_*: (e.g. *PxylP*). Partial deletions, mutations or gene-fusions are indicated superscript, dashes separate different loci. Genotype: In-frame fusions of elements are indicated by a single colon (e.g. *grxD*:*venus*), deletion of amino acids (aa) from position x to position y (if protein is not full length) are indicated with superscript delta aax-y (e.g. *grxD*^*Δaa2-19*^), point mutations, in which x at position y is exchanged by z are indicated by superscript xyz (e.g. *grxD*^*C191S*^), colons indicate gene-disruption of x by y (e.g. *pksP*::*ptrA*), delta and colons indicate replacement of x by y (e.g. *sreAΔ*::*ptrA*). Different elements of the same transformation construct are divided by a comma (e.g. *hph*,*grxD*). The genotype starts with the recipient strain followed by a semicolon (;).
(DOCX)

S3 Table Oligonucleotides used in this study. Overlaps (for NEBuilder) and restriction sites are highlighted by spaces. Mismatches (for site-directed mutagenesis) are indicated by lower-case characters.
(DOCX)
