# Supplementary figures and images for: The monothiol glutaredoxin GrxD is essential for sensing iron starvation in Aspergillus fumigatus
Source: PLoS Genet. 2019 Sep 16;15(9):e1008379. doi: 10.1371/journal.pgen.1008379 (PMC6762210; doi:10.1371/journal.pgen.1008379)

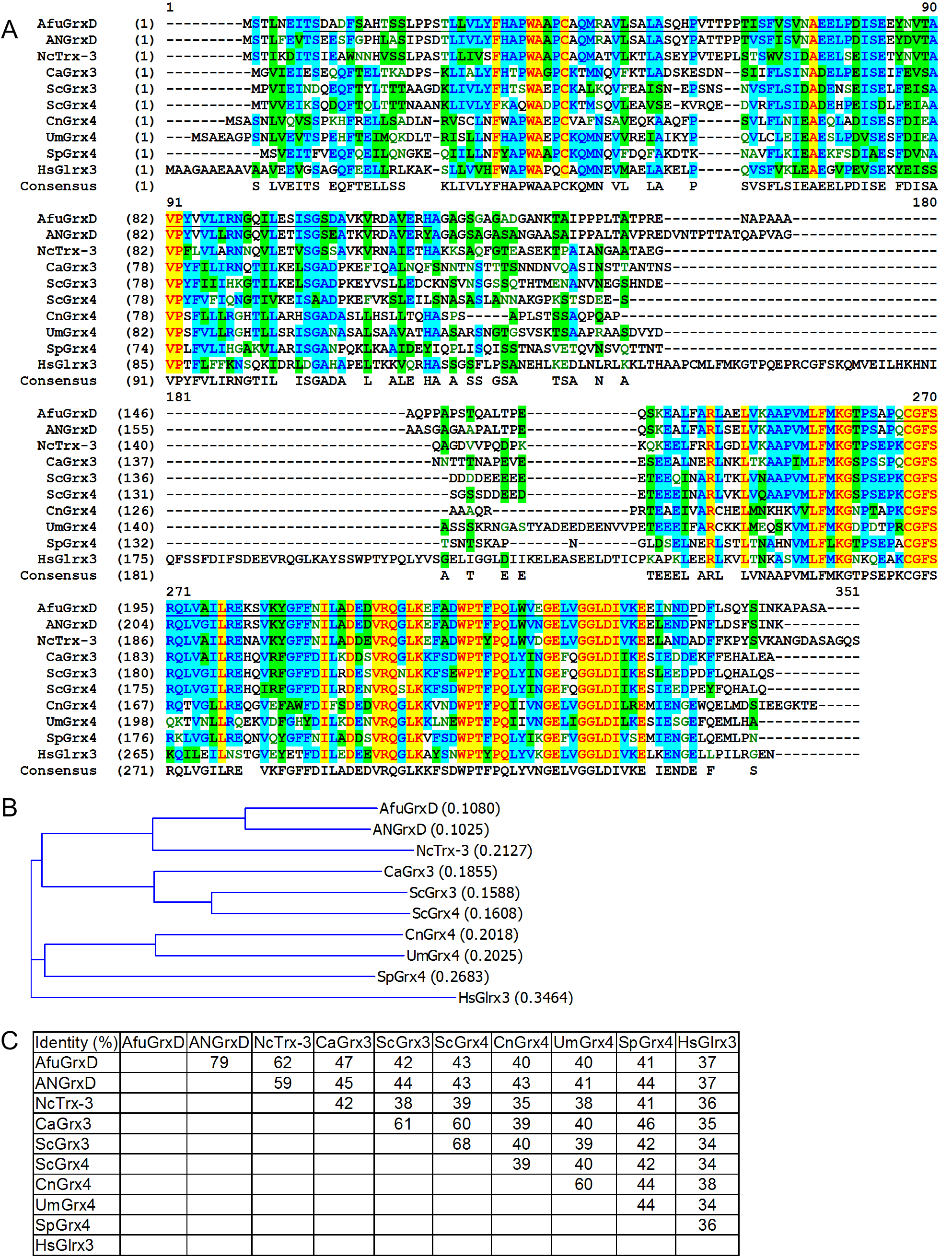

Supplement: S1 Fig — (A) Alignment of fungal and human GrxD homologs from A. fumigatus (Afu), Aspergillus nidulans (AN), Neurospora crassa (Nc), Candida albicans (Ca), S. cerevisiae (Sc), Cryptococcus neoformans (Cn), Ustilago maydis (Um), S. pombe (Sp), and Homo sapiens (Hs). The Trx-like and Grx domains of AfuGrxD are underlined. Identical residues are marked in yellow, residues conserved in 50% of the sequences are shaded in light blue and blocks of similar residues are marked in green. (B) Phylogenetic tree and (C) Identity table of the aligned amino acid sequences. Numbers in parentheses display the calculated distance values between the sequences. The multiple alignment was performed with AlignX (Vector NTI Advance 11). (TIF) [file pgen.1008379.s001.tif]

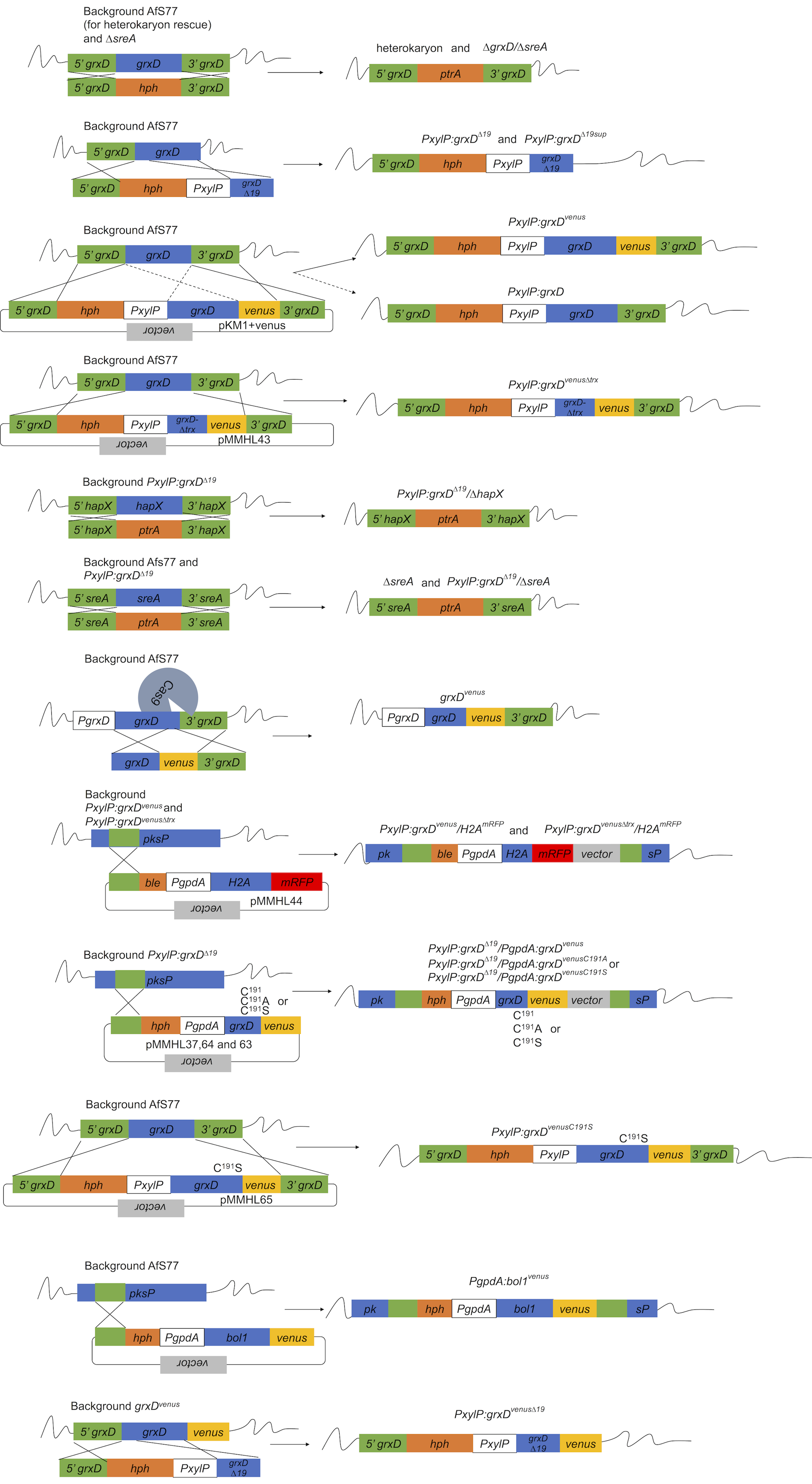

Supplement: S2 Fig — Recipient strains, genetic loci and transformation constructs employed are shown at the left; resulting strains and genetic loci are shown at the right. (TIFF) [file pgen.1008379.s002.tiff]

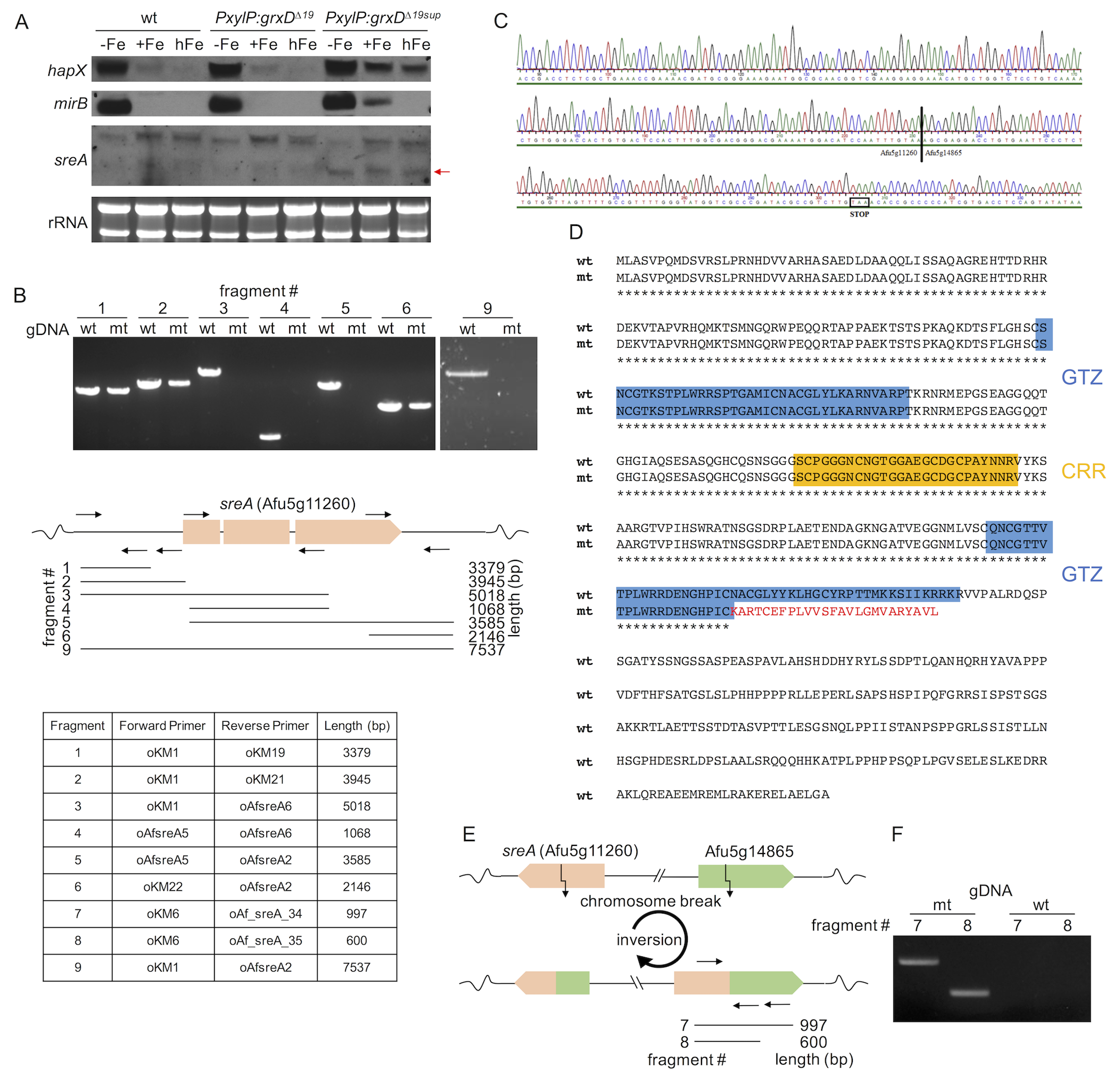

Supplement: S3 Fig — (A) Northern analysis of hapX and mirB in wt, PxylP:grxDΔ19, and PxylP:grxDΔ19sup strains under iron starvation (-Fe), iron sufficiency (+Fe) and high iron conditions (hFe) under 0.1% xylose inducing conditions. The additional sreA transcript in PxylP:grxDΔ19sup is indicated by a red arrow (B) PCR-amplification analysis demonstrating a recombination in the genomic sreA locus: agarose gel electrophoresis, strategy for PCR-amplification of the sreA locus and primers employed. The failing PCR amplification of fragments 3, 4, 5, and 9 (entire locus) from genomic DNA of strain PxylP:grxDΔ19sup (mt) compared to wt indicated a breakpoint in exon 1 or 2. (C) Sequence analysis of the amplicon obtained by 3´-RACE from strain PxylP:grxDΔ19sup, using sreA specific primers located in the sreA 5´-UTR, revealed a chimeric mRNA containing the 5´-end of the sreA transcript and the 3´-end of the transcript encoded by Afu5g14865. (D) Alignment of wt SreA and the deduced amino acid sequence of the chimeric cDNA obtained by 3´-RACE (mt). This analysis revealed chromosomal recombination within the second GATA-type zinc finger (GTZ; boxed in blue)-coding region of SreA, which caused SreA inactivation. Identical amino acids are indicated by asterisks; differences in the deduced chimeric amino acid sequence are shown in red; CRR (cysteine-rich region) is boxed in yellow. (E) Scheme of the chromosomal rearrangement in PxylP:grxDΔ19sup resulting in inactivation of SreA. (F) PCR-amplification analysis (agarose gel electrophoresis) of the sreA locus of PxylP:grxDΔ19sup (mt) compared to wt proving the inversion. (TIFF) [file pgen.1008379.s003.tiff]

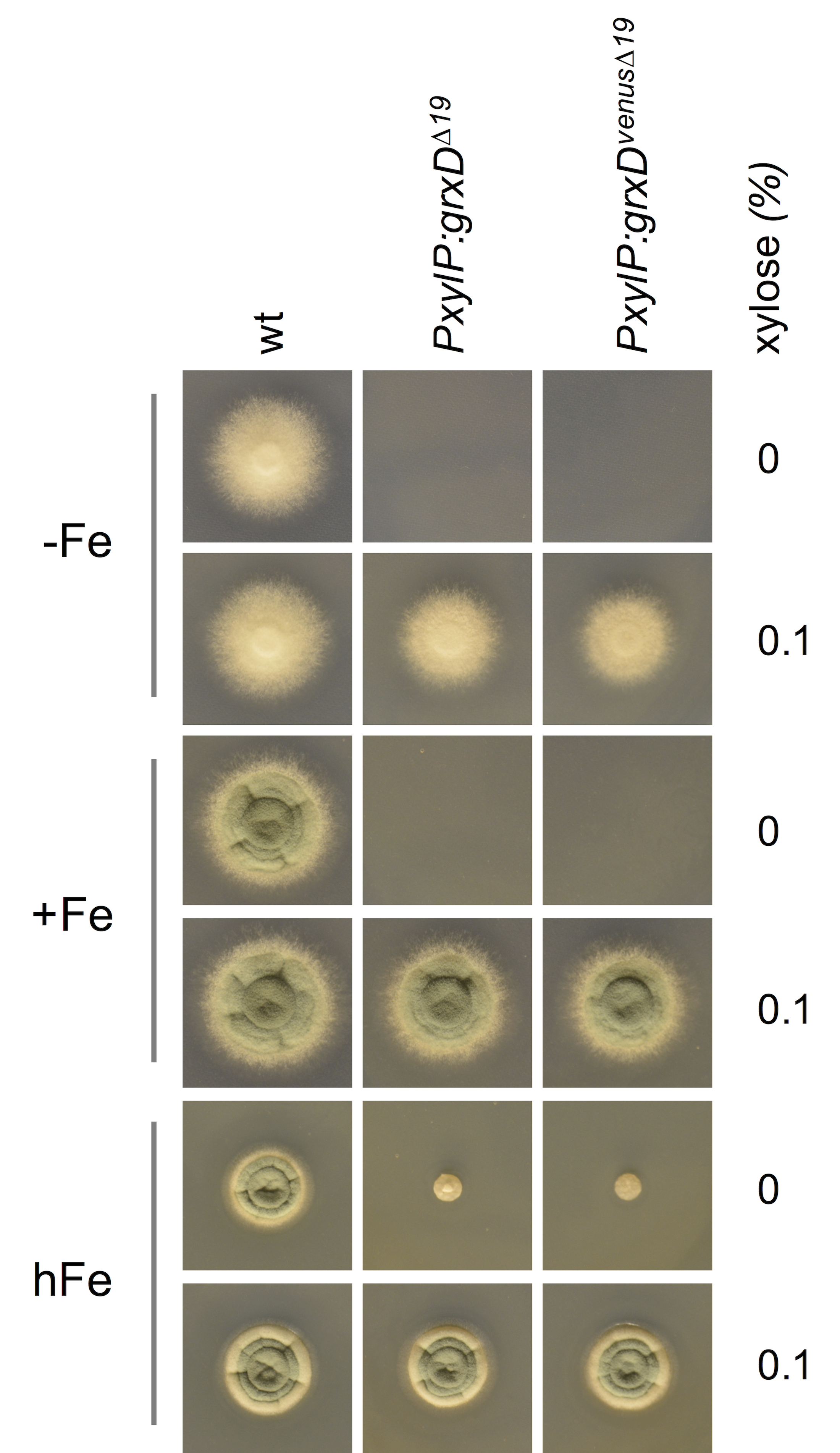

Supplement: S4 Fig — Strains were grown for 48 h at 37°C in minimal medium under non-inducing (0% xylose) and inducing (0.1% xylose) conditions with iron starvation (-Fe), iron sufficiency (+Fe) and iron excess (hFe), respectively, as described in Fig 2. (TIFF) [file pgen.1008379.s004.tiff]

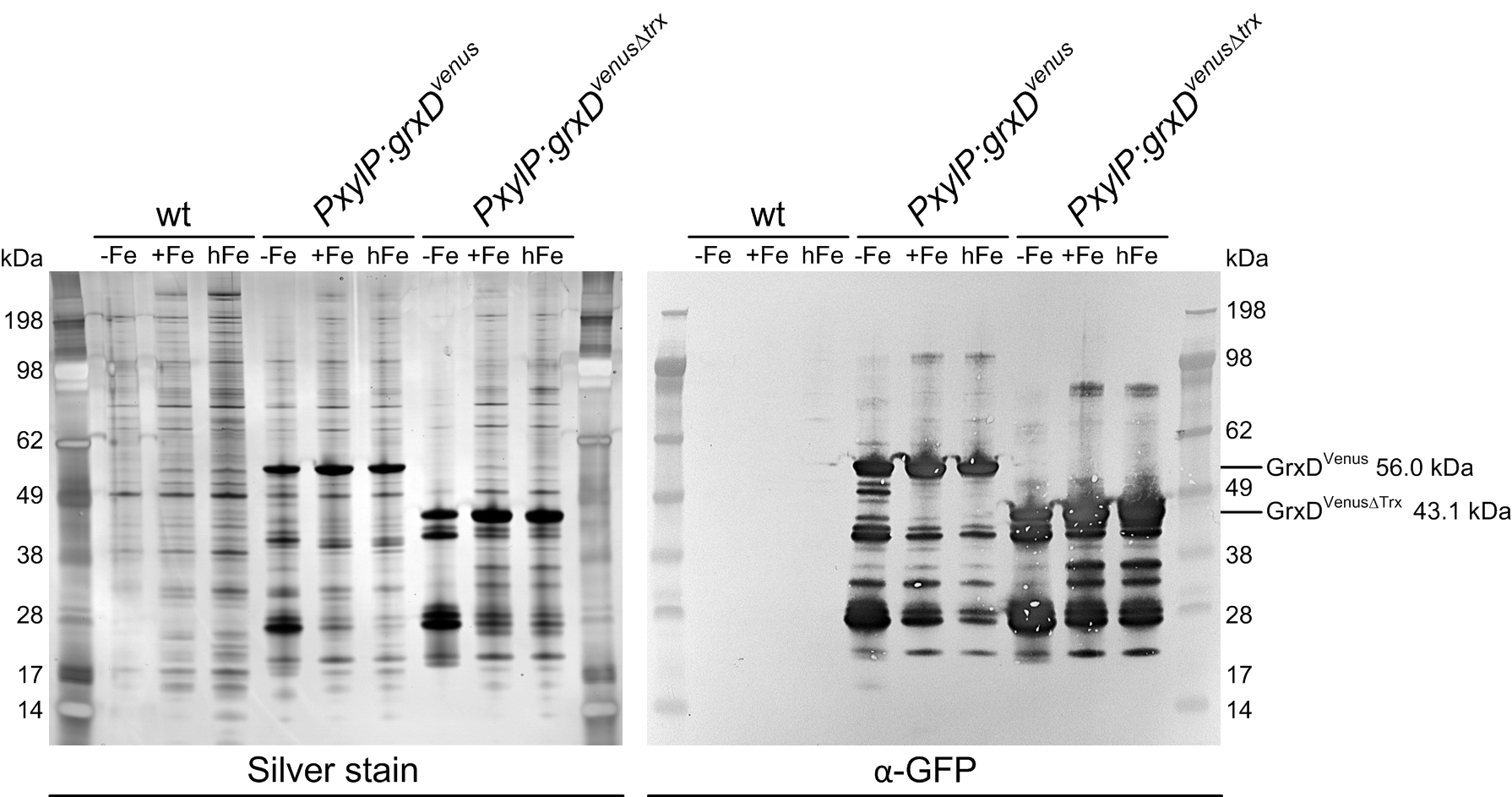

Supplement: S5 Fig — (TIF) [file pgen.1008379.s005.tif]

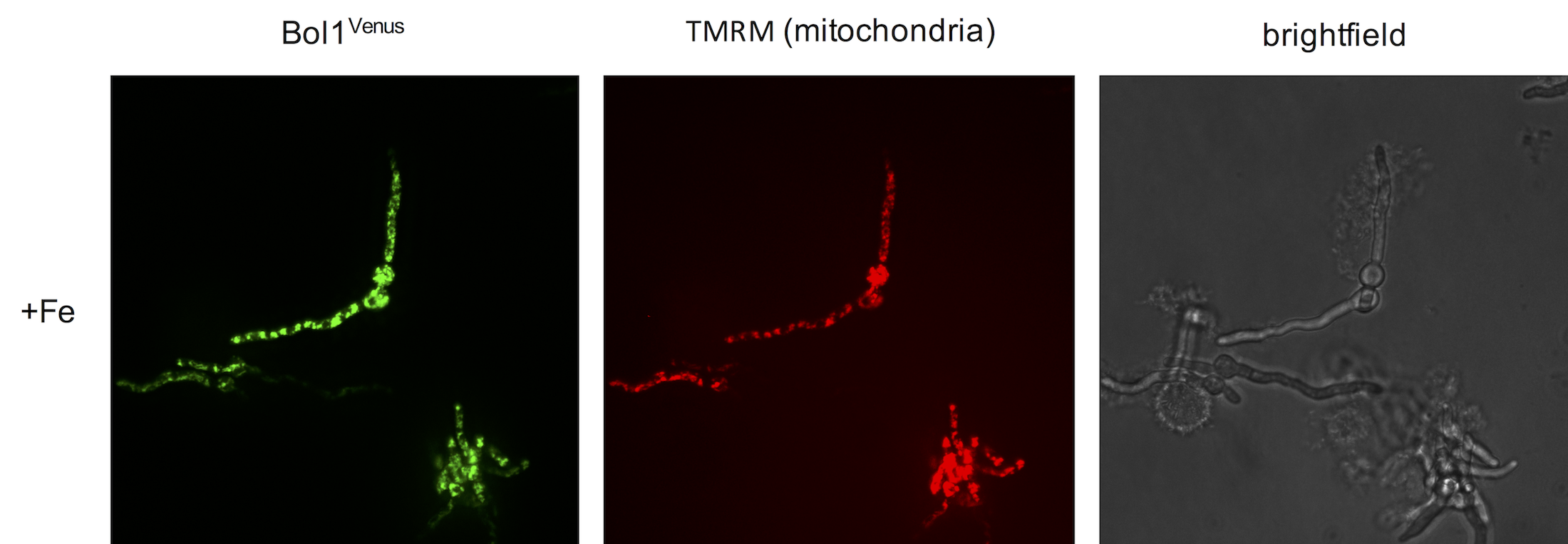

Supplement: S6 Fig — For fluorescent microscopy, strain PgpdA:bol1venus was grown for 18 h in minimal medium. To visualize mitochondria, the mitochondria specific dye tetramethylrhodamine (TMRM) was used. (TIFF) [file pgen.1008379.s006.tiff]

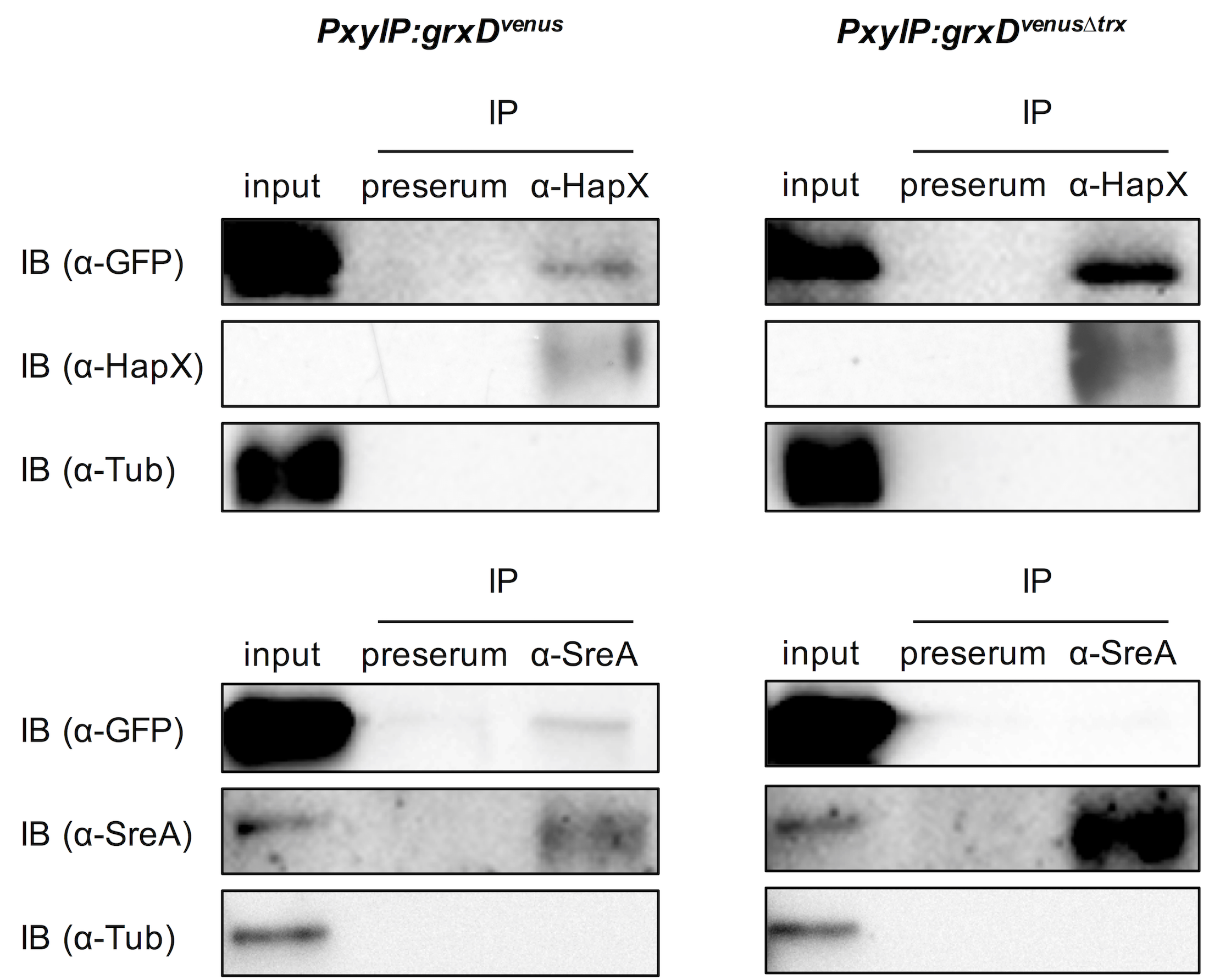

Supplement: S7 Fig — HapX and SreA, respectively, were immunoprecipitated with indicated antisera (IgGs covalently linked to Protein-A-Sepharose) in cell free protein extracts obtained from Venus-tagged GrxD or GrxDΔTrx producing strains PxylP:grxDvenus or PxylP:grxDvenusΔtrx, respectively, grown for 16 h in 0.1% xylose containing minimal medium without iron supplementation for HapX or 0.03 mM iron supplementation for SreA. Immunoprecipitates (IP) were analyzed for Co-IP of GrxDVenus or GrxDVenusΔTrx by immunoblot analysis (IB) with a mouse α-GFP antibody. Successful precipitation of HapX respectively SreA was analyzed by IB analysis with rabbit α-HapX or rabbit α-SreA antisera. HapX levels in the input were below the detection limit. Tubulin was used as a loading control. (TIFF) [file pgen.1008379.s007.tiff]

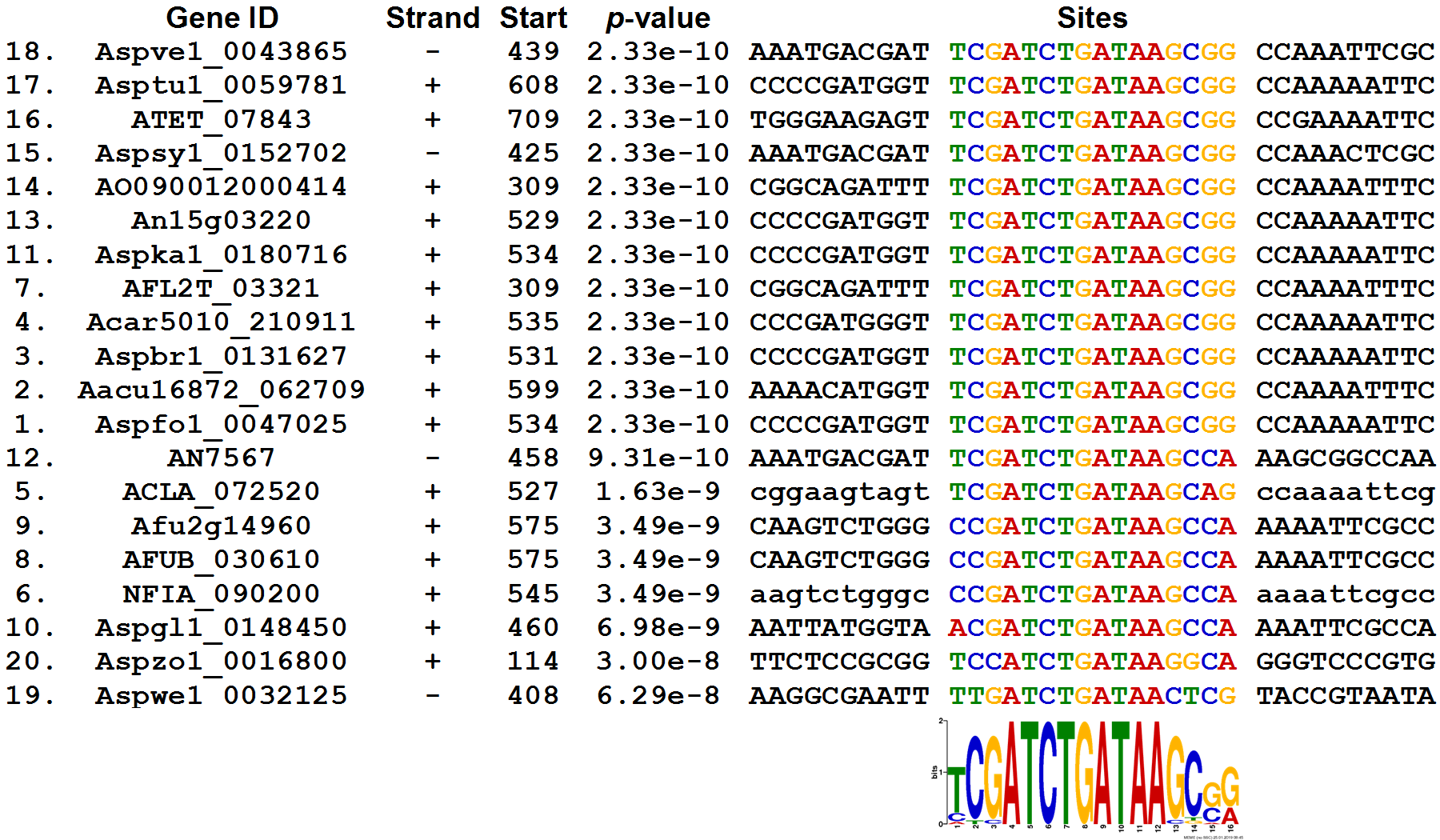

Supplement: S8 Fig — MEME motif 1 in PgrxD of 20 Aspergillus spp. (SreA target motif 5´-ATCWGATAA-3´). For promoter analysis, the complete 5´ intergenic non-coding grxD regions were selected. Putative transcription factor motifs were identified using the MEME motif discovery tool provided by the MEME suite platform. The following parameters were used: motif width 6–16 bp; zero or one occurrence per sequence. In the first ranked motif 20 sites were counted with an E-value of 1.0e-067. (TIF) [file pgen.1008379.s008.tif]

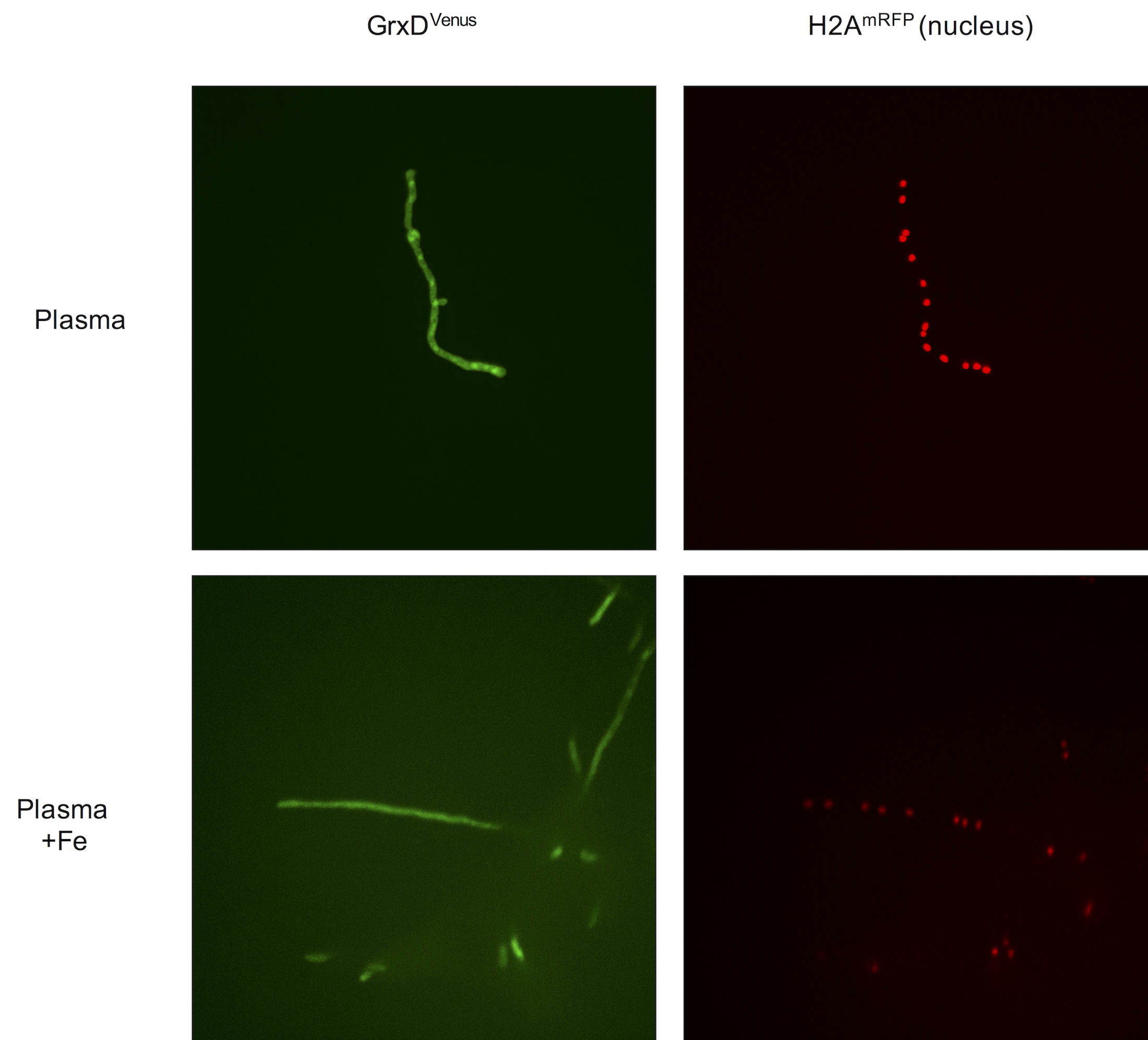

Supplement: S9 Fig — For fluorescent microscopy, strain PxylP:grxDvenus/H2AmRFP was grown for 18h with 0.05% xylose under iron starvation (-Fe) or iron sufficiency (+Fe). The mRFP-tagged histone H2A served to visualize nuclei. (TIFF) [file pgen.1008379.s009.tiff]

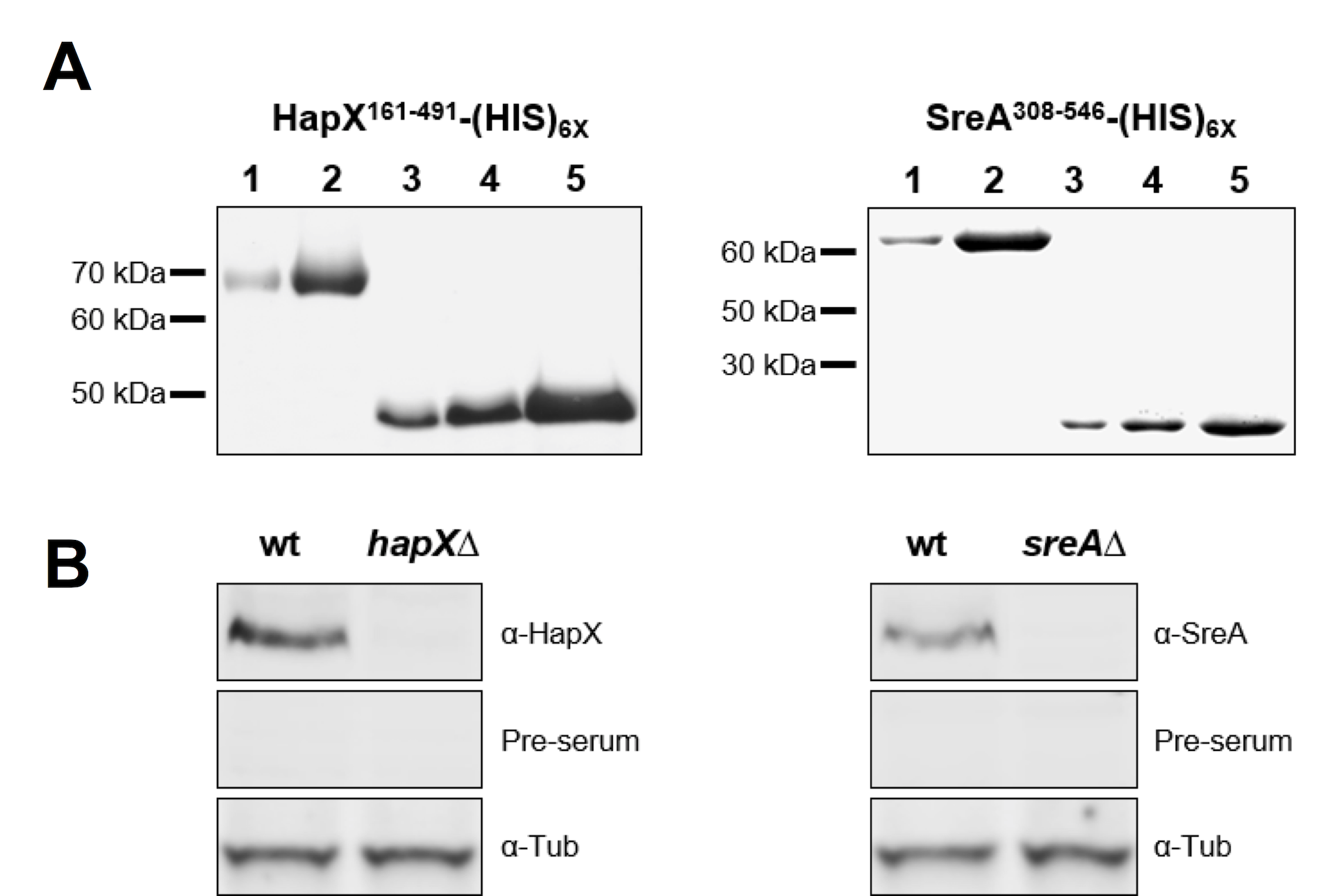

Supplement: S10 Fig — (A) Coomassie-stained gels of HapX161-491-(HIS)6X and SreA308-546-(HIS)6X polypeptides after purification (see materials and methods). Lanes 3, 4 and 5 of each gel show the amount of protein in 2.5, 5 and 10 μl. 1 and 5 μg of BSA were loaded as controls in lanes 1 and 2, respectively. (B) Western blot analysis with rabbit α-HapX or rabbit α-SreA antisera, and their respective pre-sera as negative controls. Strains were grown in -Fe (for α-HapX blot) and +Fe (for α-SreA blot) minimal medium for 20 h at 37°C. α-Tubulin was used as loading control. (TIFF) [file pgen.1008379.s010.tiff]
