# Supplementary material for: The monothiol glutaredoxin GrxD is essential for sensing iron starvation in Aspergillus fumigatus
Source: PLoS Genet. 2019 Sep 16;15(9):e1008379. doi: 10.1371/journal.pgen.1008379 (PMC6762210; doi:10.1371/journal.pgen.1008379)
Supplement: S2 Table — For Stains: Δ indicates loss of gene-function, exchanged promoters are indicated with P_: (e.g. PxylP). Partial deletions, mutations or gene-fusions are indicated superscript, dashes separate different loci. Genotype: In-frame fusions of elements are indicated by a single colon (e.g. grxD:venus), deletion of amino acids (aa) from position x to position y (if protein is not full length) are indicated with superscript delta aax-y (e.g. grxDΔaa2-19), point mutations, in which x at position y is exchanged by z are indicated by superscript xyz (e.g. grxDC191S), colons indicate gene-disruption of x by y (e.g. pksP::ptrA), delta and colons indicate replacement of x by y (e.g. sreAΔ::ptrA). Different elements of the same transformation construct are divided by a comma (e.g. hph,grxD). The genotype starts with the recipient strain followed by a semicolon (;). (DOCX) [file pgen.1008379.s012.docx]

**S8 Table.**

| **Strain** | **Genotype** | **Reference** |
| --- | --- | --- |
| wt | AfS77 | [1] |
| *∆hapX* | AfS77*; hapX∆::ptrA* | [2] |
| *∆sreA* | AfS77*; sreA∆::ptrA* | this study |
| *grxD^venus^* | AfS77*; grxD:venus* | this study |
| *PxylP:grxD* | AfS77*; grxD∆::hph,PxylP:grxD* | this study |
| *PxylP:grxD^venus^* | AfS77*; grxD∆::hph,PxylP:grxD:venus* | this study |
| *PxylP:grxD^∆19^* | AfS77*; grxD∆::hph,PxylP:grxD^∆aa2-19^* | this study |
| *PxylP:grxD^∆19sup^* | AfS77*; sreA::chromosomal rearrangement* | this study |
| *PxylP:grxD^venus∆trx^* | AfS77*; grxD∆::hph,PxylP:grxD^∆aa2-125^:venus* | this study |
| *PxylP:grxD^∆19^/∆sreA* | *PxylP:grxD^∆19^; sreA∆::ptrA* | this study |
| *PxylP:grxD^∆19^/∆hapX* | *PxylP:grxD^∆19^; hapX∆::ptrA* | this study |
| *∆grxD/∆sreA* | *AfS77; sreA∆::ptrA; grxD∆::hph* | this study |
| *PxylP:grxD^venus^/H2A^mRFP^* | *PxylP:grxD^venus^; pksP::ble,PgpdA:mRFP:H2A* | this study |
| *PxylP:grxD^venus∆trx^/H2A^mRFP^* | *PxylP:grxD^venus∆trx^; pksP::ble,PgpdA:mRFP:H2A* | this study |
| *PxylP:grxD^∆19^/PgpdA:grxD^venus^* | *PxylP:grxD^∆19^; pksP::ptrA,PgpdA:grxD:venus* | this study |
| *PxylP:grxD^∆19^/PgpdA:grxD^venusC191A^* | *PxylP:grxD^∆19^; pksP::ptrA,PgpdA:grxD^C191A^:venus* | this study |
| *PxylP:grxD^∆19^/PgpdA:grxD^venusC191S^* | *PxylP:grxD^∆19^; pksP::ptrA,PgpdA:grxD^C191S^:venus* | this study |
| *PxylP:grxD^venusC191S^* | AfS77*; grxD∆::hph,PxylP:grxD^C191S^:venus* | this study |
| *PxylP:grxD^venus∆19^* | AfS77; *grxD∆::hph,PxylP: grxD^∆aa2-19^:venus* | this study |
| *PgpdA:bol1^venus^* | AfS77; *pksP::ptrA,PgpdA:bol1:venus* | this study |

**References:**

[1] Hartmann T, Dümig M, Jaber BM, Szewczyk E, Olbermann P, Morschhäuser J, et al. Validation of a self-excising marker in the human pathogen Aspergillus fumigatus by employing the beta-rec/six site-specific recombination system. Appl Environ Microbiol 2010;76:6313–7. doi:10.1128/AEM.00882-10.

[2] Gsaller F, Hortschansky P, Beattie SR, Klammer V, Tuppatsch K, Lechner BE, et al. The Janus transcription factor HapX controls fungal adaptation to both iron starvation and iron excess. EMBO J 2014;33:2261–2276.
