# Supplementary material for: The monothiol glutaredoxin GrxD is essential for sensing iron starvation in Aspergillus fumigatus
Source: PLoS Genet. 2019 Sep 16;15(9):e1008379. doi: 10.1371/journal.pgen.1008379 (PMC6762210; doi:10.1371/journal.pgen.1008379)
Supplement: S3 Table — Overlaps (for NEBuilder) and restriction sites are highlighted by spaces. Mismatches (for site-directed mutagenesis) are indicated by lower-case characters. (DOCX) [file pgen.1008379.s013.docx]

**S9 Table**

| **Name** | **Sequence** |
| --- | --- |
| oAfgrx4-oe1 | ATCCGTGTTCCTTGCGAG |
| oAfgrx4-oe2 | CAT CCTAGG TGAGAGAGAATGTCGCGG |
| oAfgrx4-oe3 | TTCTTGAACCTCCTCGCC |
| oAfgrx4-oe4 | CCT CCATGG CTCCCTCCACCTTACTAG |
| oAfgrx4-oe5 | GCTCAGGAAATCAGGGTC |
| oAfgrx4-oe6 | GAAATGTAGGCCAGTCCG |
| oAfgrx4-oe7 | GAG TCTAGA TATGCCAGATGACGCCAG |
| oAfgrx4-oe8 | AGGATGGCAACCAACTGG |
| oAfgrx4-1 | TTCGCCGTCAACTCTCTTCTT |
| 3' RACE Outer Primer | GCGAGCACAGAATTAATACGACT |
| 3' RACE Inner Primer | CGCGGATCCGAATTAATACGACTCACTATAGG |
| 3' RACE Adapter | GCGAGCACAGAATTAATACGACTCACTATAGG-T_12_-VN |
| oAf-pksP1-f | ATGCAAGCTT CATCAGTGGTGGTGTAAACC |
| oAf-pksP2-r | ATGCAAGCTT TGAAGACGAACCCAGTCTTG |
| oAfhapX-1 | AGCGACTATAGCCGGATG |
| oAfhapX-2 | CCTTGGGTCTTGAAGCTTGCG |
| oKM11 | AATTCGAGCTCGGTAC GTCGTCTGCCGCTTG |
| oKM12 | TACCTA GG GGTGCTTGAGAGAGAAT |
| oKM13 | CAAGCACC CCTAGGTACAGAAGTCC |
| oKM14 | GTCGACAT GGTTGGTTCTTCGAGT |
| oKM15 | AACCAACC ATGTCGACGCTCAAC |
| oKM16 | GCCAAGCTTGCATGCC GGATTCCACATGGATCT |
| oKM26 | TGTACAAG TGAAGGTACGACGGG |
| oKM27 | ACGGATGA AGCGCTGGCAGGAGC |
| oKM28 | CCAGCGCTT CATCCGTAACCATGG |
| oKM29 | TACCTTCA CTTGTACAGCTCGTCC |
| oMM156 | ACAAGTGA CCATGGCAGCAGTGA |
| oMM157 | GTCGACAT TGTGATGTCTGCTCAAG |
| oMM158 | ACATCACA ATGTCGACGCTCAAC |
| oMM159 | TGCCATGG TCACTTGTACAGCTCGT |
| oMM164 | AATTCGAGCTCGGTAC AAAGCTTCTACTTACTAGCA |
| oMM165 | AATCAATT CGCCACTATCAACCC |
| oMM166 | TAGTGGCG AATTGATTACGGGATCCC |
| oMM167 | ACTGCGCA TCTTGCATCTTTGTTTGTAT |
| oMM168 | ATGCAAGA TGCGCAGTCCCTTCA |
| oMM169 | GCCAAGCTTGCATGCC CCTGCGCACGCTGCT |
| oMM182 | AAGACGGCTATCCCTCCT |
| oMM184 | CATGGTTGGTTCTTCGAGTCG |
| oMM189 | AGTGAATTCGAGCTCGGTAC CCTAGGTACAGAAGTCCAATTG |
| oMM190 | GGCAAAGATC TCTAGAAAGAAGGATTACCTC |
| oMM191 | TCTTTCTAGA GATCTTTGCCCGGTGTATG |
| oMM192 | GATAGGGAGA ATATCTGGAGGGGACAGG |
| oMM193 | CTCCAGATAT TCTCCCTATCGCCAGCAA |
| oMM194 | TTACGCCAAGCTTGCATGCC GAGAATCGGGTCCACTTG |
| oMM301 | AGTGAATTCGAGCTCGGTAC GTCGTCTGCCGCTTGTGATTTTG |
| oMM302 | TGTACCTAGG GGTGCTTGAGAGAGAATGTCG |
| oMM303 | CTCAAGCACC CCTAGGTACAGAAGTCCAATTG |
| oMM304 | ACCCGTCGTACCT TCTAGAAAGAAGGATTACCTC |
| oMM305 | TCTTTCTAGA AGGTACGACGGGTGCTCTTG |
| oMM306 | TTACGCCAAGCTTGCATGCC GGATTCCACATGGATCTTATGTACATG |
| oMM313 | TGCACCACAGtcgGGATTCAGTC |
| oMM314 | CTAGGTGTACCCTTCATG |
| oMM315 | TGCACCACAGgcaGGATTCAGTCG |
| oMM356 | TCATCCGTAACCATGGTC |
| oMM357 | TGTGATGTCTGCTCAAGC |
| oMM358 | CCGCTTGAGCAGACATCACA ATGCTCCCCATCCGCAGC |
| oMM359 | CTGACCATGGTTACGGATGA AGCCTGCGACTGTCTAATCTTC |
| AfgrxDNdeI126f (ΔTrx) | AACTGCATATGAAAACGGCGATTCCGCCACCGTTG |
| AfgrxDBamHIr (ΔTrx) | TTGACGGATCCAGCGCTAGCCGGAGCTTTGTTGATG |
| AfgrxDC191Af | CCCAGTGCTCCGCAGgcgGGCTTTTCTCGC |
| AfgrxDC191Ar | GCGAGAAAAGCCcgcCTGCGGAGCACTGGG |
| AfhapX161-491C203Af | GCTGGGTTGTAATGATgcgAGCACCAGCCATTGTCAG |
| AfhapX161-491C203Ar | CTGACAATGGCTGGTGCTcgcATCATTACAACCCAGC |
| AfhapX161-491C277Af | CCGGCAGTTGATCCGgcgGGTTTTTGTAGTGATGGC |
| AfhapX161-491C277Ar | GCCATCACTACAAAAACCcgcCGGATCAACTGCCGG |
